# Polymer Electrolytes for Supercapacitors

**DOI:** 10.3390/polym16223164

**Published:** 2024-11-13

**Authors:** Xuecheng Chen, Rudolf Holze

**Affiliations:** 1 Faculty of Chemical Technology and Engineering, West Pomeranian University of Technology, Szczecin, Piastów Ave. 42, 71-065 Szczecin, Poland; 2 Confucius Energy Storage Lab, School of Energy and Environment, Southeast University, Nanjing 210096, China; 3 Department of Electrochemistry, Institute of Chemistry, Saint Petersburg State University, 7/9 Universitetskaya Nab., St. Petersburg 199034, Russia; 4 Chemnitz University of Technology, D-09107 Chemnitz, Germany; 5 State Key Laboratory of Materials-Oriented Chemical Engineering, School of Energy Science and Engineering, Nanjing Tech University, Nanjing 211816, China

**Keywords:** supercapacitor, capacitive storage, electrolytes, polymer electrolytes, solid electrolytes, gel electrolytes

## Abstract

Because of safety concerns associated with the use of liquid electrolytes and electrolyte solutions, options for non-liquid materials like gels and polymers to be used as ion-conducting electrolytes have been explored intensely, and they attract steadily growing interest from researchers. The low ionic conductivity of most hard and soft solid materials was initially too low for practical applications in supercapacitors, which require low internal resistance of a device and, consequently, highly conducting materials. Even if an additional separator may not be needed when the solid electrolyte already ensures reliable separation of the electrodes, the electrolytes prepared as films or membranes as thin as practically acceptable, resistance may still be too high even today. Recent developments with gel electrolytes sometimes approach or even surpass liquid electrolyte solutions, in terms of effective conductance. This includes materials based on biopolymers, renewable raw materials, materials with biodegradability, and better environmental compatibility. In addition, numerous approaches to improving the electrolyte/electrode interaction have yielded improvements in effective internal device resistance. Reported studies are reviewed, material combinations are sorted out, and trends are identified.

## 1. Introduction

**Figure 1:**
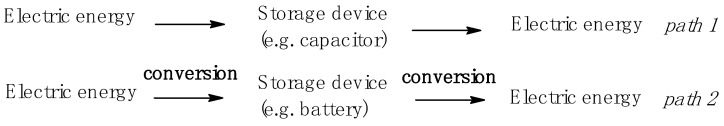
Schematic pathways of electric energy storage EES.

Supercapacitors have established themselves as superior high-power devices for the storage of electric energy without any transformation or conversion (see Figure 1, path 2) encountered by batteries or electrolyzers and fuel cells (see Figure 1, path 1).

They provide large currents and high power; unfortunately, their energy density is still inferior to that of secondary batteries (contrary statements in a report fraught with numerous errors are apparently unfounded [1]). But they avoid the typical drawbacks of secondary batteries associated with electrode reactions, material transformations (see Figure 1, path 2), and further details, which severely limit the stability and lifetime of secondary batteries. A typical supercapacitor contains two highly porous electrodes on metal foils as current collectors, with a thin, porous layer of a separator in between. The whole setup is soaked with an aqueous or non-aqueous electrolyte solution (for an overview, see [2]; a rather mysterious contribution can be found in [3]). This phase certainly does not store charge as presumably suggested in a fundamental misunderstanding in [4,5,6]. Obviously, the arrangement is very similar to that of a secondary battery; actually, a supercapacitor may be called an extreme version of a high-power battery, whereas a secondary battery may be considered as a high-energy version of a supercapacitor. This merger between these initially very different operational principles has been reviewed elsewhere [7]. Not surprisingly, some of the problems faced by secondary batteries show up with the supercapacitors again. Liquid electrolytes and liquid electrolyte solutions are among them. The drawbacks of using such liquids in batteries have been addressed frequently; many users have actually encountered such drawbacks with leaking aqueous systems spilling corrosive alkaline liquid once the battery container has been perforated by corrosion (even the steel containers sometimes employed do not withstand the corrosive attack of the cell ingredients forever) or by mechanical damage. With organic solvent-based electrolyte solutions in lithium and lithium-ion batteries, further dimensions like flammability, evaporation, and toxicity were added. With aqueous electrolyte solutions being preferable for many reasons, the narrow voltage limit for device operation with electrolyte decomposition of water is a further weakness. Consequently, the search for other non-liquid options meeting some or all of the indicated flaws has been active in battery research, and now it is also active in supercapacitor research and development [2].

Solid electrolytes based on ion-conducting inorganic crystalline or amorphous materials (glasses, ceramics, etc.) are one option, and organic polymers providing ionic conductance are another one [8]. Apparently, only the latter materials should be called solid polymer electrolytes SPE whereas, e.g., porous films or membranes of a polymer soaked with some ionic liquid (see, e.g., [9,10]) or electrolyte solution (see, e.g., [11]) should be more precisely called a separator or a modified separator. The sometimes-noticed opinion that anything that is not liquid should be called a solid may be an oversimplification; it is definitely confusing. This confusion is possibly equivalent to the designation “quasi-solid-state” [12,13,14]^1^. For even more impressive linguistic developments toward a “pseudo-solid-state electrolyte”, see [15]; toward a “quasi-solid-state supercapacitor”, see [5,11,16,17,18]; toward a “quasi-solid polymer electrolyte”, see [19,20]; and toward a “quasi-solid-state hybrid battery supercapacitor”, see [21]. Anyway, the terminology has been confused and remains confusing; for more examples, see [22,23]. A remarkably confusing example is contained in a report wherein a mixture of polyvinylidene fluoride (PVDF) with an ionic liquid has been designated a solid electrolyte [24]. Another example of confusion is found in [25] wherein, e.g., an “ionic-liquid-embedded polymer electrolyte” is mentioned. The material is simply an ionogel. A helpful, but unfortunately not commonly accepted, designation of materials as “solid-like electrolytes” has been suggested [26]. Mixtures of several polymers are commonly called polymer blends, and mixtures of inorganic and organic materials (not necessarily polymers) are sometimes called hybrids—in particular, when beneficial extra effects (not simply additive ones) of the components are observed as studied in, e.g., [27].

Unfortunately, the ionic conductivity values of most solids are still rather low, in particular at room temperature. This might not be a major problem with, e.g., low current batteries like those employed in pacemakers, which have been using a solid layer of LiI formed between the lithium metal negative electrode and the positive electrode of iodine and poly-2-vinylpyridin successfully for years [28]. With supercapacitors and their very large current capabilities being their most prominent feature, this is a very serious problem. Nevertheless, some progress has been made with organic polymeric electrolytes; this will be reviewed in the following. When a suitable liquid is added to a polymer, it might turn into a gel; the obtained material may be called a gel(led) polymer electrolyte (GPE). Given the open question of where to place a gel [29,30], this option shall not be overlooked here; it will be addressed further in Section 3.6 of this report.

A major problem of most supercapacitors is the self-discharge that proceeds much faster than in batteries [2,31,32]. Because energy storage in the currently (at the time of this writing) dominant electrochemical double layer capacitors (EDLC) is based on charge separation (discharge proceeds in the opposite direction by charge, i.e., ion, dissipation), self-discharge will always simply proceed once the device is charged, by spontaneous redistribution of ions. A barrier slowing down this process will also slow down self-discharge. Because self-discharge is directly related to ion movement, the poor conductivity of solid electrolytes of every type related to low ion mobility and a low mobile charge carrier concentration may also suggest a slower self-discharge. Although in the vast majority of the research reports inspected for this overview, self-discharge is not even mentioned (data on self-discharge are even more rare); sometimes, it is addressed as in [33]. In this report, we notice a remarkably slower self-discharge as compared to a corresponding device with a liquid electrolyte solution; this improvement does not come at the price of compromised performance (for details, see below).

The addition of redox-active ingredients to the electrolyte solution for increased storage capability is a frequently discussed option [2]; it has also been examined with polymer electrolytes and will be discussed in Section 3.7 below.

Solid electrolytes may finally ensure sufficient separation of both electrodes without an additional separator. This advantage is sometimes explicitly mentioned; frequently, the absence of a separator can only be inferred from the present or missing details of the experimental description.

In this review, an attempt is made to provide a complete inspection of all research papers found with the search string specified in more detail in the following section. Characteristic details of every material/electrolyte/practical solution are provided, enabling the reader to assign the entry to a particular class of electrolytes as tentatively suggested in this review. Finally, the reader should be able to find a class of electrolyte (i.e., in a section below) or even a specific material fitting in their own research or a promising and most suitable electrolyte. To avoid later frustration in terms of insufficient durability, lifetime data are provided whenever available. Further data on electrolytes are not given because they will be hardly comparable or compatible between various reports and will be highly incomplete anyway.

## 2. Fundamentals: Ionically Conducting Polymers

First reports about ionically conducting polymers, i.e., polymeric solid electrolytes for EES, dealt with poly (ethylene oxide) (PEO, (-CH_2_O-)_n_), poly (acrylonitrile) (PAN), poly (methyl methacrylate) (PMMA) and some other heteroatom-containing polymers. Their ionic conductivity at common operating temperatures was too low for practical application [34,35]. Thus, further materials like polymers with ionogenic functional groups (sulfonates, carboxylates, etc.) capable of releasing a proton or the initially mentioned polymers with electrolytes dissolved into them have been developed [22]. The acronym SPE^2^ (solid polymer electrolyte) was proposed; nowadays, it is mostly applied to cation exchange membranes (CEM, Nafion^®^, Flemion^®^) in electrolyzers and fuel cells. Their application in supercapacitors will be reviewed in Section 3.3.

**Figure 2:**
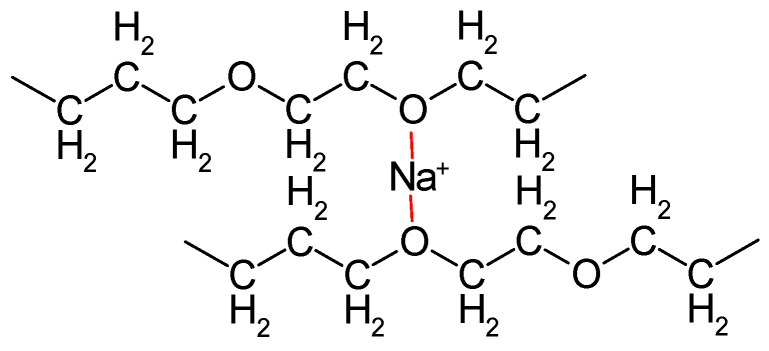
Scheme of interactions between a sodium ion and PEO.

The disadvantage of the low ambient temperature conductivity of polymer electrolytes (about 1/100 or even 1/1000 of the ionic conductivity of inorganic conductors based on ceramics, glasses, or inorganic crystals) is more than compensated by their advantages; in particular, the option of preparing very thin but mechanically still stable films for wound cell designs, and the usually lower energy demand in preparation. The polar groups (in particular those containing oxygen in, e.g., PEO) interact with ions of the dissolved salt (Figure 2). Thus, SPEs may be considered as solvents. The molecular architecture of an SPE, chain flexibility, and further details are relevant for actually observed conductivity [34]; for overviews, see also [26,36]. The influence of segmental motion in polymer chains of a plasticized PEO-electrolyte on ion transport has been examined [37].

**Figure 3:**
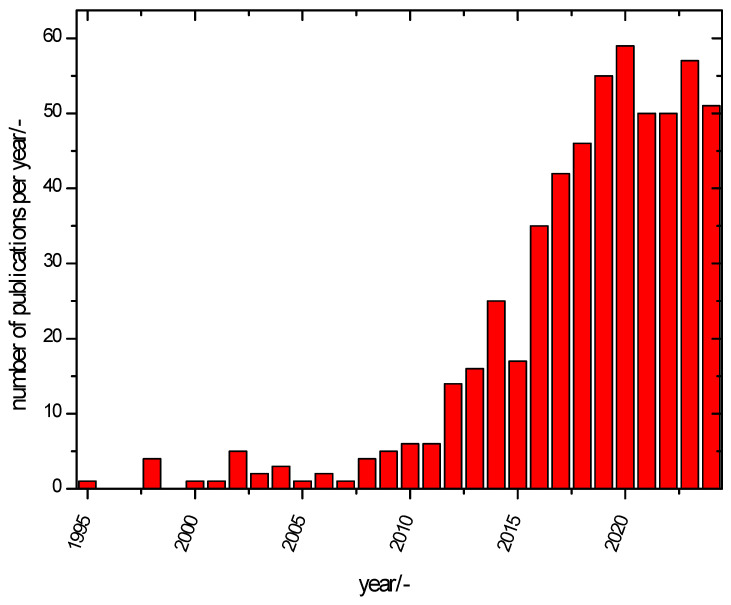
Annual publication numbers of reports with “polymer electrolyte” and “supercapacitor” anywhere in the title, keywords, or abstract (Data from Scopus^®^ and Web of Science^®^ retrieved on 8 July 2024). Further publications with these keywords somewhere in the text could not initially be counted, but when noticed and considered relevant in the present context, they were evaluated below. The very few publications on “electrochemical capacitors” or “double layer capacitors” instead of “supercapacitors” were included; the associated confusion suggests once more systematic use of technical terms. The term “polymer electrolyte” is repeatedly mentioned in the abstract but nowhere addressed in the report; this might have resulted in numbers slightly too large.

The development of research activities, starting with initial reports and reviewed in [34], also becomes apparent when looking at publication activities, as displayed in Figure 3.

In the sections of the following main chapter, reports on polymer materials studied as electrolytes in supercapacitors are reviewed and organized according to the class of polymer and further functional criteria already outlined above. Following the already addressed confusing terminology in subsequent sections, materials not exactly fitting into this classification are inspected in the section appearing most fitting or suggested by the author(s). Although sometimes synthesis and characterization of electrolytes appear to be the main purpose of a report, applications in actual devices are central to this report. For reasons stated elsewhere [38] and keeping in mind the deplorable absence of common standards for reporting specific capacitances and energy values [39], such values are not reported here. Instead, values indicative of stability are stated (when provided) in terms of capacitance retention as a function cycle number because capacitance retention as a function of time of use or of run charge/discharge cycles will ultimately decide the success of a device in the market. Changes in power density (whether volumetric or gravimetric ones) or energy density (again volumetric or gravimetric ones) as a function of cycle number are rarely reported. Unfortunately, in the noteworthy exception [40], an EDLC device has been cycled only 250 times! Thus, the following capacitance values, i.e., their retention, are the focus of attention. Although ionic conduction appeared as the focal point of all studies and developments, its actual specific value for a given material is only one dimension of the studied materials. Critically important is the actual Ohmic resistance in a practical cell (as part of the electric series resistance ESR). In the case where the mechanical properties of a given material make a thinner electrolyte foil possible, even low specific conductivity may become less important. If the material, in addition, supports better interfacial contact between the porous electrode and the electrolyte, this aspect will be even less dominant. The requirements for an electrolyte, in this report a polymer electrolyte, can be summed up:

- Wide available electrode potential window;
- High ionic conductivity and sufficient chemical and electrochemical stability;
- Thermal stability;
- Compatibility with electrode and separator materials;
- Environmental compatibility;
- Low price;
- Sustainable resources.

The role of electrolytes has been mentioned in passing in a boisterous report on the “emerging electrochemical activation tactic” [41]. Actually, the authors discuss changes in cell constituents during the operation of the cell, and they observed their effects!

Overviews beyond the already mentioned monographs [34,35] on SPEs touching fundamentals and many fields of applications are available [13,22,42,43,44,45,46,47]. They include reviews focused on specific details like processability [48], the identity of the mobile charge carriers like OH^−^-ions in [49], influence of stretching on conductivity [50], safety aspects of GPEs [51] and further general aspects of GPEs [52], processing of GPEs [53], inkjet printability of GPEs [54], SPEs in flexible supercapacitors [55,56,57,58], SPEs combined with MXenes [59], self-healing SPEs [60], proton-conducting polymers with particular attention to their blends with inorganic materials [61], cellulose-based SPEs [62], and radiation-grafting in SPE preparation [63]. In the following text, the acronym SPE will be used mostly without addressing again and again the details of gelling, etc.

## 3. Solid Polymer Electrolytes in Supercapacitors

### 3.1. Plain SPEs

A polymer with ionic substituent groups enabling the release of mobile ions by dissociation and supporting the movement of the released ions by some hopping or migration mechanism can be called a polymer electrolyte; the acronym SPE is most frequently associated with these materials only. The adjective solid is only an addition; it is actually redundant because polymers tend to be solid. Because of this ion-releasing capability, these materials will be treated in Section 3.3.

Actually, the development of polymer electrolytes started with materials without the former features like PEO, PAN, PMMA, NYLON^®^, and further heteroatom-containing polymers. Precisely speaking, considering the definition of the term electrolyte [2], these materials should not be called electrolytes. Their ionic conductivity, which is only based on their capability to support ion movement without releasing or creating ions at common operating temperatures, was too low for practical application [34]. This might have been due to the number of mobile charge carriers being too low and their mobility being too slow. At least two roads toward improvement appeared: Chemical modification of a polymer by insertion of ionogenic groups and combination of polymers with plasticizing substances were options pursued subsequently (see following section). Nevertheless, these materials have been further studied as host materials for electrolytes.

#### 3.1.1. Polyvinylidene Difluoride

An electrospun polyvinylidene difluoride (PVDF) membrane was soaked in a solution of an IL in an unknown solvent and used as SPE in a redox supercapacitor keeping 93% of its initial capacitance after 100 (!) cycles [64]. PVDF dissolved in propylene carbonate with dissolved LiClO_4_ left a gel after solvent evaporation and was subsequently used as an electrolyte in a hybrid supercapacitor with reduced graphene oxide and manganese oxide electrodes [65]. A SPE based on this material combination was used in a hybrid supercapacitor with a Ni(OH)_2_-reduced graphene oxide showing 67% capacitance retention after 5000 cycles [66]. The use of this SPE for direct printing of both EDLC devices and redox supercapacitors has been demonstrated [67]. In a similar approach, PVDF was dissolved in a mixture of ethylene and propylene carbonate; after the addition of NaSCN, an SPE was obtained and tested in a symmetric redox supercapacitor with PANI electrodes [68]. Stability was not examined. The authors repeated the study with PPy as electrode material and examined stability along 50 (!) cycles [69]. PVDF and chitosan dissolved in a solvent mixture of aqueous acetic acid and DMF yielded an SPE for a symmetric redox supercapacitor, showing unusual increases and subsequently decreases in capacitance with cycling [70].

PVDF and an IL dissolved in acetone yielded an SPE subsequently used in an EDLC device with unknown stability [71]. PVDF dissolved in DMF with an added IL and nanoparticular SiO_2_ has been tested in an EDLC device, which showed 9% capacitance loss after 2000 cycles [72].

#### 3.1.2. Polyurethane

Polyurethane plasticized with a mixture of ethylene and propylene carbonate with added LiClO_4_ as an ion source has been used in an EDLC device, which kept 80 of its initial capacitance after 1000 cycles [73].

#### 3.1.3. Polyacrylates

An SPE of poly (lithium acrylate) with silica nanoparticles has been reported; the text addition of LiTFSI^3^ is mentioned later [74]. An EDLC device kept 97% of its initial capacitance after 12,000 cycles. Sodium acrylate copolymerized with lignin and nanoparticular SiO_2_ afforded an SPE for an EDLC device with 96% capacitance retention after 8000 cycles [75]. A copolymer of vinylimidazole and hydroxypropyl acrylate with added NaNO_3_ was used as self-healing SPE in an EDLC device showing a stable capacitance during 5000 cycles [76].

Several acrylate-related SPEs have been compared in a redox supercapacitor with polyaniline electrodes; the device with polyacrylic acid and sulfuric acid as SPE showed the most stable capacitance values during 5000 cycles (assuming the labeling of the respective Figure to be wrong with a correct labeling given in the report) [77].

**Figure 4:**
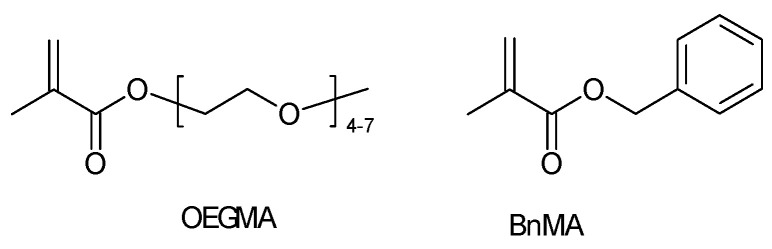
(Ethylene glycol) methyl ether methacrylate and benzyl methacrylate.

A “methacrylate-based” SPE not specified further^4^ has been suggested for use in high-power electrochemical storage and conversion devices [78]. Inspection of possibly related publications by the authors of this report leads to [79]. Again, the use of “methacrylate-based” electrolytes for lithium-ion batteries is claimed in the title. The polymer is actually a statistical copolymer of oligo(ethylene glycol) methyl ether methacrylate (OEGMA) and benzyl methacrylate (BnMA) (see Figure 4).

Discs punched off the obtained SPE membranes were soaked in a solution of LiPF_6_ in a mixture of ethylene carbonate and dimethyl carbonate. Possibly, this SPE was also used in supercapacitor studies in [78]. The assembled EDLC device lost about 10% of the initial capacitance after 50,000 cycles. For a lithium-ion capacitor, capacitance retention depended on current density; at lower current densities, losses were more pronounced than at higher current densities.

SPEs based on poly (2-ethoxyethyl methacrylate) plasticized with various carbonate solvents and with added ILs as ion sources have been compared [80]. A proton-conducting SPE based on 2-hydroxyethyl methacrylate with further ingredients was tested in an EDLC device, yielding an optimum composition of the SPE 96% capacity retention after 1000 cycles [81].

#### 3.1.4. Poly (Ethylene Glycol)

Cross-linked poly (ethylene glycol) dimethacrylate and poly (ethylene glycol) methyl ether methacrylate with an added IL have been prepared as SPEs for an EDLC device with a capacitance stable along 2500 cycles [82]. Why this material was called poly ethylene oxide in the title of the report remains mysterious.

A microporous membrane of poly (ethylene glycol)–grafted poly (arylene ether ketone) filled with a chitosan-based aqueous LiClO_4_ gel electrolyte was examined in an EDLC device [83]. Capacitance was stable along 5000 cycles.

An SPE based on a copolymer of poly (ethylene glycol) methyl ether acrylate (PEGMA) and trimethylolpropane ethoxylate triacrylate formed in a solution of ethylene carbonate and dimethyl carbonate with LiPF_6_ was used in a microsupercapacitor keeping 83% of its initial capacitance after 5000 cycles [84].

**Figure 5:**
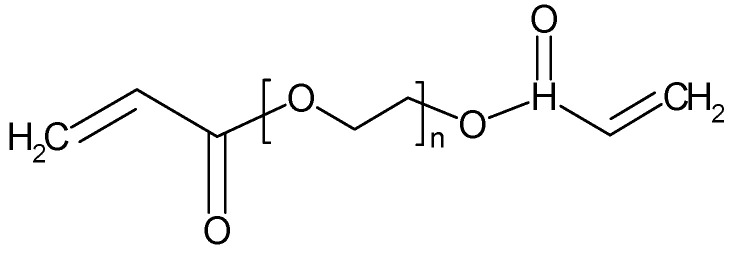
Polyethylene glycol diacrylate.

Polyethylene glycol diacrylate (Figure 5) combined with various Ils has been used in the preparation of microsupercapacitor array, which yields a device keeping its capacitance along 20,000 cycles [85]. Polyethylene glycol diacrylate was photopolymerized in the presence of Mg(TFS)_2_ and succinonitrile as plasticizer and used as SPE in an EDLC-device^5^ keeping 87% of its initial capacitance after 11,200 cycles [86]. An SPE of polyethylene glycol diacrylate with LiBF_4_ as an ion source, an IL, and SiO_2_ nanoparticles have been prepared and characterized [87].

**Figure 6:**
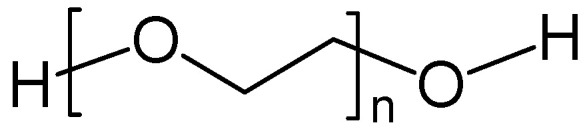
Poly (ethylene glycol).

A blend of poly (ethylene glycol) (Figure 6) and chitosan plasticized with ethylene and propylene carbonate with LiClO_4_ as an ion source was used as SPE in an EDLC device providing 91% capacitance retention after 1000 cycles [88].

An injectable type of SPE based on poly (ethylene glycol) methyl ether methacrylate with good penetration into the porous carbonaceous electrodes of the EDLC device finally assembled was tested with poor capacitance retention at elevated operation temperature *T* = 80 °C, data at room temperature were not provided [89]. Several acrylate-related monomers were combined into a copolymer used in an EDLC device [90]. After assembly, the device was soaked in an electrolyte solution of spiro-(1,10)-bipyrrolidinium tetrafluoroborate in acetonitrile. Specific capacitance was larger than found with the electrolyte solution instead of the SPE. This confirms a very good utilization of the internal surface of the porous electrode; an 85% capacitance retention after 5000 cycles confirms this assumption.

#### 3.1.5. Nylon^®^

A blend of Nylon^®^ 6–10 with H_3_PO_4_ has been proposed as an SPE for an EDLC-supercapacitor [91,92].

#### 3.1.6. Poly (ethylene oxide)

PEO mixed with an ionic liquid and dissolved in acetone was poured onto a porous polypropylene membrane separator [9]. The assembly of an EDLC device was not described; how sufficient contact between electrode and electrolyte was established remains mysterious. After 1000 cycles, 90% of the initial capacitance was retained. To a solution of PEO in methanol, an IL was added; the SPE obtained after solvent evaporation was tested in an EDLC device without the obtained stability data [93].

PEO cross-linked with an IL and benzophenon fixed in a non-woven separator yielded an SPE tested in an EDLC device with unspecified stability [94]. PEO combined with an aqueous solution of KOH yielded an SPE in an EDLC device with unknown stability [95]. Correlations between ion transport and further properties and stretching of a PEO-IL SPE have been studied [96]. To PEO dissolved in propylene carbonate, an IL was added, the obtained SPE was tested in an EDLC device, and stability was not examined [97].

In a redox capacitor, an SPE based on organo nanoclay, Et_4_NBF_4_, and PEO was used [98]. The device lost 31% of its initial capacitance during 1000 cycles.

An SPE prepared from PEO, organically modified nanoclay, and tetraethylammonium tetrafluoroborate has been prepared and characterized [99]. The performance of the assembled EDLC device at room temperature was poor; stability was not examined.

An application of an electrolyte composed of PEO, LiTFSI, and the ionic liquid *N*-methyl-*N*-propylpiperidinium bis(trifluoromethansulfonyl)imide has been reported [100]. PEO modified by electron beam irradiation has been studied as an SPE [101]. Although no supercapacitors were assembled, specific capacitances were surprisingly measured. The observed increase with irradiated PEO suggests a beneficial effect of increased conductivity of the polymer. To a PEO-based solid electrolyte with NaPF_6_ nanoparticles of ZrO_2_ were added, the stability of the assembled EDLC-supercapacitor was not examined [102]. To PEO with NH_4_I as an ion source, carbon black has been added as a filler for improved electrochemical properties (increased ionic conductivity) at very low concentrations 0.01 to 0.06 wt.% [103]. The reduced charge transfer resistance also attributed to added fillers is not addressed anywhere in the report, and the stability of the assembled EDLC device was not examined.

A PEO/PVP/LiTFSI/BaTiO_3_ electrolyte for various applications in electrochemical energy storage has been reported [104]. A mixture of PEO and PVA with LiClO_4_ as an ion source as SPE has been studied [105].

#### 3.1.7. Polyacrylamide

An SPE based simply on poly (2-acrylamido-2-methyl-1-propanesulfonic acid) has been used in an EDLC device, keeping more than 80% of its initial capacitance after 5000 cycles [106]. A neutral SPE based on polyacrylamide and Li_2_SO_4_ has been developed and tested in an EDLC device, which showed a stable capacitance during 10,000 cycles [107].

#### 3.1.8. Polysulfone

A cross-linked sulfonated poly (ether ether ketone) has been used as SPE in a redox supercapacitor with PANI as active masses in both electrodes [108,109]. For improved electrolyte/electrode contact, the assembly was soaked in sulfuric acid. It showed about 15% capacitance loss during 1000 cycles. Sulfonated polysulfone with added boric acid, an IL, and polyphosphoric acid has been used as SPE in an EDLC device, showing 85% capacitance retention after 1000 cycles [110].

#### 3.1.9. Copolymers

The formation of copolymers in their various forms [111] has been considered as an option to improve the relevant properties of polymer electrolytes. Copolymers with IL are presented in Section 3.4. A poly (ethylene oxide)-poly (propylene oxide)-poly (ethylene oxide) triblock polymer further cross-linked with 200% added IL has been used as an SPE in an EDLC device showing 95% capacitance retention after 10,000 cycles [112]. The significant IL content, which was called somewhat surprisingly “moderate” in the report, enabled penetration into the porous electrodes accounting for the impressive rate capability. Copolymers of polyacrylic acid with other comonomers have been prepared and combined with NaNO_3_ from an aqueous solution, yielding a hydrogel used in an EDLC device showing 89% capacitance retention after 3000 cycles [113]. A polymethacrylate comb copolymer combined with an IL has been used in an EDC device, which kept 91% of its initial capacitance after 10,000 cycles [114]. Organically modified ceramics, i.e., inorganic-organic copolymers, have been introduced [115].

SPEs of poly (acrylic acid)-co-poly (acrylamide) copolymer and the corresponding homopolymer poly (acrylic acid) with added KCl have been compared in a symmetric redox supercapacitor with MnO_2_-based electrodes [116]. In terms of capacitance retention, the cell with the copolymer SPE performed much better, showing no capacitance loss within 3000 cycles. This was tentatively attributed to the higher rigidity of the copolymer, which was indicated by a much higher glass transition temperature.

A poly (aryl ether ketone)-poly (ethylene glycol) copolymer has been tested as a host material with added LiClO_4_ in supercapacitors at elevated temperatures [117]. The EDLC-type electrodes were soaked with dimethylacetamide before assembly. The electrolyte membrane was prepared by dissolving the copolymer in dimethylacetamide followed by the addition of various amounts of LiClO_4_ (The reference to EO, presumably ethylene oxide, in the report appears to be a mistake). Upon assembly of the supercapacitor, some salt moves into the liquid, staying in the porous electrode body, which helped establish a sufficiently extended electrode/electrolyte interface. The recorded capacitance stayed almost stable at *T* = 30 °C and 120 °C during 2000 cycles. A poly (ether ether ketone)/poly (vinyl alcohol) composite membrane has been suggested as a separator (not an SPE) for an EDLC device with an aqueous electrolyte solution [118].

A poly (arylene ether sulfone) copolymer membrane has been examined in an EDLC device [119]. Soaking with a solution of Li_2_SO_4_ (presumably in water) yielded an SPE, enabling a stable capacitance along 3000 cycles. An apparently similar material called a copolymer with again no identification of the second comonomer with polyether side chains has been studied again as SPE elsewhere by these authors [120]. To an amphiphilic block-graft copolymer poly (styrene-*b*-butadiene-*b*-styrene)-*g*-poly (oxyethylene methacrylate) propylene carbonate and LiTFSI were added [121]. The somewhat hard-to-understand experimental description suggests that a solution of this polymer, presumably in THF, was dropped onto the porous activated carbon electrode. Apparently, the dried electrodes were assembled into a supercapacitor without a separator. About 5% of the initial capacitance was lost after 2000 cycles. How PEO side chains were formed remains a mystery.

**Figure 7:**
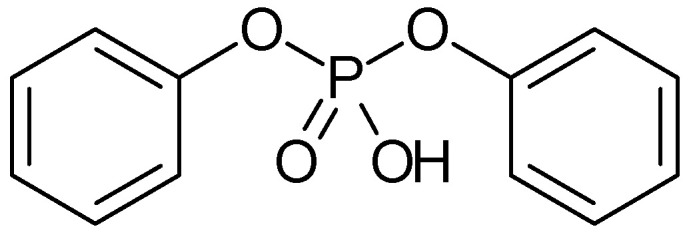
Diphenyl phosphate.

A copolymer of methyl methacrylate and 2-hydroxyethyl methacrylate combined with diphenyl phosphate (Figure 7) as a proton source has been used as SPE without stability data reported [122].

A copolymer poly (hydroxyethyl methacrylate-co-trimethylolpropane allyl ether) combined with H_3_PO_4_ was used as an SPE in an EDLC device, which showed an 84% capacitance retention after 32,000 cycles [123].

A copolymer of 2-hydroxyethyl methacrylate and [2-(acryloyloxy)ethyl]-trimethylammonium chloride was swollen in highly concentrated phosphoric acid, yielding a SPE tested in a symmetric redox supercapacitor with polyaniline electrodes [124]. The device showed a stable capacitance of up to 9000 cycles. Subsequently, a serious drop in capacitance to 86% of the initial value at 11,000 cycles was observed.

A copolymer of poly (2,2,2-trifluoroethyl methacrylate) and poly (ethylene glycol) behenyl ether methacrylate with an added IL was used as SPE in an EDLC device showing 88% capacitance retention after 4000 cycles [125]. With a copolymer of poly (isobornyl methacrylate) and poly (ethylene glycol) methyl ether methacrylate used as SPE, an EDLC device provided an initially growing capacitance, which retained around 90% after 6000 cycles [126]. A copolymer of acrylonitrile and vinyltrimethoxysilane was soaked with an electrolyte solution of LiPF_6_ in ethylene carbonate/dimethyl carbonate and used as SPE in a hybrid supercapacitor with unknown stability [127]. The copolymer poly (vinyl alcohol-co-acrylonitrile) was combined with PEO and an IL into an SPE for an EDLC device; somewhat surprisingly, the SPE without PEO performed best in stability and energy density [128]. A microporous polymer membrane of a similar polymer soaked with an aqueous solution of 1 M LiClO_4_ has been called a polymer electrolyte [129]; the device prepared with an optimized membrane (the terminology appears to be somewhat misleading; actually, the membrane serves as a separator and does not have any of the typical functions of an electrolyte) showed a slightly higher specific capacity than a device with liquid electrolyte and decreased self-discharge. The small amount of added chitosan is presumably the reason for calling the membrane a gelled electrolyte in a figure. A sodium polyacrylate-co-polyacrylamide SPE has been compared with the respective homopolymer sodium polyacrylate SPE with KCl as an ion source in a redox supercapacitor [130]. The copolymer provided much superior capacitance retention during 3000 cycles; this was attributed to the alkaline nature of the amino groups of the comonomer.

A porous silica network-derived poly (styrene)-b-poly (2-vinylpyridine) block copolymer filled with an IL has been suggested as an SPE for an EDLC device, which showed 76% capacitance retention after 1000 cycles [131].

A hydrogel composed of three comonomers, one hydrophilic and two hydrophobic ones, with LiCl, has been proposed with the aim of improving low-temperature and high-voltage performance [132]. The assembled EDLC device kept 90% of its initial capacitance after 10,000 cycles.

A complex mixture of PTFE, polyurethane, fumed silica nanoparticles, and an ionic liquid yielded an electrolyte called polymeric by the authors for an EDLC device with a capacitance stable along 5000 cycles [133]. The aging of the electrolyte and the electrolyte/electrode interface were not examined. A copolymer of potassium poly (acrylate) and polyurethane soaked with an aqueous solution of Na_2_SO_4_ was used as a SPE in a redox supercapacitor with 97% capacitance retention after 10,000 cycles [134]. A composite of polyurethane and porous wood with polyethylene glycol, 2,2-bis(hydroxymethyl)propionic acid, and LiClO_4_ as an ion source has been used in an EDLC device with 95% capacitance retention after 4000 cycles at optimum composition [135].

A copolymer of vinyl acetate and 1-ethyl-3-vinylimidazolium with the anion bromide of the latter as a mobile charge carrier has been tested as SPE with a wide electrochemical window of stability in an EDLC device with 90% capacitance retention after 5000 cycles [136]. Cross-linked poly (acrylic acid-co-vinylimidazole) soaked in LiCl-containing solutions in water and/or ethylene glycol had been tested in an EDLC device; no stability data were communicated [137]. For further examples of copolymer-based SPEs, see, e.g., [138]. A copolymer electrolyte, namely poly (*N*-isopropylacrylamide-co-glycidyl methacrylate), which showed a steep decrease of ionic conductivity and almost caused a shutdown of a supercapacitor, has been proposed to provide 88% capacitance retention after 1000 cycles in a MXene-based device [139]. A copolymer of three monomers poly (acrylonitrile)-b-poly (ethylene glycol)-b-poly (acrylonitrile) has been swollen with DMF; LiClO_4_ was added as an ion source [140]. For the assembled EDLC device, stability was not reported.

#### 3.1.10. Epoxy-Based Polymers

Epoxy resin loaded with a high fraction of the IL DMIMBr yielded a flexible electrolyte [141]. Epoxy combined with poly (ethylene glycol) containing a mixture of an IL and LiTFSI formed a double network electrolyte, enabling 61% capacitance retention after 3000 cycles [142]. An epoxy polymer prepared in a solution of an IL with dissolved LiTFSI as an ion source with added Al_2_O_3_ for improved conductivity and mechanical robustness has been used as an SPE in an EDLC device with 61% capacitance retention after 1000 cycles [143]. An SPE employing an epoxy-based polymer as host material with a solution of LiTFSI and an IL as the embedded ion-conducting phase has been tested in an EDLC device with stability not reported [144]. An epoxy-based polymer rich in polyethylene oxide moieties was used as a host for an IL; the product was used as an SPE in a fiber supercapacitor (EDLC-type) with stability not reported [145]. The advantages of epoxy-based SPEs have been highlighted in [146]; a review of these SPEs is available [147].

#### 3.1.11. Polyester

An SPE containing polyester, a lithium salt, an IL, and PANI nanofibers has been developed [148]. Although the mixture was infused into the porous electrode before curing for better electrode/electrolyte contact, the displayed data suggest a rather high internal resistance of the assembled device; the device kept 93% of its initial capacitance after 2500 cycles. The same electrolyte was applied with different electrode materials by the same authors [149]. This device kept 96% after 2000 cycles. Nitrile butadiene rubber soaked with an aqueous solution of KCl has been used as SPE in a redox supercapacitor with unknown stability [150].

#### 3.1.12. Chemically Modified Biopolymers

Chemically modified methylcellulose with LiTFS as electrolyte salt has been examined as an electrolyte in an EDLC device with less than 4% capacitance lost after 20,000 cycles [151]. For improved electrolyte/electrode interfacing, the still liquid electrolyte material was poured on the porous electrodes. Methylcellulose mixed with NH_4_NO_3_ and an IL yielded an SPE with unknown stability in an EDLC device [152]. A repetition of this study with a mysterious MC electrolyte material (with MC identified as methylcellulose by a careful comparison of both publications) yielded an EDLC device showing a fluctuating capacitance within 180 (!) cycles [153]. Methylcellulose with various amounts of propylene carbonate as plasticizer and NaI as an ion source (why the author called it a dopant remains a mystery) has been tested in an EDLC device [154]. Stability data were not given. Apparently, the iodide ions were not electractive in the studied cell voltage range. An SPE prepared from cellulose acetate with anadded IL and KSCN as ion source has been prepared and characterized; stability data of an assembled EDLC device were not reported [155].

LiClO_4_ was added as an ion source to functionalized methyl cellulose; the obtained SPE was tested in an EDLC device showing less than 5% capacitance losses after 30,000 cycles [20]. An SPE from hydroxy ethylcellulose with KOH was used in an EDLC device showing 91% capacitance retention after 10,000 cycles [156]. A carboxymethylcellulose^6^-based SPE with Na_2_SO_4_ as an ion source has been used in a hybrid supercapacitor showing 80% capacitance retention after 10,000 cycles [157]. A SPE based on carboxymethylcellulose intercalated with plant particles of dried *Hibiscus sabdariffa*^7^ and citric acid as an ion source was used as SPE in an EDLC device keeping 91% of its initial capacitance after 4000 cycles [158].

Lignocellulose soaked with sulphuric acid has been called a hydrogel or gel polymer for reasons not entirely clear [159]. An EDLC device assembled with this electrolyte kept its initial capacitance after 20,000 cycles. These authors tried the same approach with KOH instead [160].

An SPE prepared by dissolving cellulose acetate and LiClO_4_ in THF has been prepared and tested for biodegradability [161]. A test in a redox capacitor with PPy electrodes showed a stepwise capacitance loss of about 5% after 250 cycles.

An SPE from cassava starch and H_2_SO_4_ with carbon dots added for enhanced performance was used in an EDLC device, which kept 93% of its initial capacitance after 10,000 cycles [162]. A blend of chitosan and starch plasticized with glycerol and LiClO_4_ as an ion source (not as dopant as claimed in the text) has been tested in an EDLC device of unknown stability [163]. This blend and the plasticizer combined with NaI have been used in a device whose stability is not examined [164]. Chitosan combined with a deep eutectic solvent yielded an SPE tested in a redox supercapacitor, which showed a 70% capacitance retention after 1500 cycles [165]. Chitosan combined with potato starch and graphene oxide was used as a solid electrolyte in a redox supercapacitor, with stability not reported [166]. Elsewhere, potato starch has been used in the preparation of a multicomponent electrolyte with a description too complicated to present in this overview [167]. A blend of poly (styrene sulphonic acid) and starch with added LiClO_4_ with glycerol as a plasticizer blend enabled an EDLC device with a few percent capacitance loss along 3000 cycles [168]. A xanthan-gum-based SPE with Na_2_SO_4_ has been tested as an SPE for an EDLC device, which showed an 85% capacitance retention after 3000 cycles [169]. Guar gum, with a small amount of PEDOT:PSS and LiClO_4_, enables an EDLC device with 98% capacitance retention after 1000 cycles [5]. An SPE of guar gum plasticized with glycerol and LiClO_4_ as an ion source has been prepared, characterized, and tested in an EDLC device, which kept 94% of its initial capacitance after 2000 cycles [170]. A blend of poly (caprolactone) and guar gum with LiClO_4_ as an ion source has been tested as SPE in an EDLC device, which lost about 5% of the initial capacitance during 2000 cycles [171].

PVA cross-linked with acrylic acid and xanthan gum with added ZnCl_2_ (presumably as an ion source) was used as SPE for a zinc ion capacitor [172]. The device showed 84% capacitance retention after 1000 cycles.

An SPE prepared from chitosan and adipic/acetic acid, an ionic liquid, and a lithium salt has been prepared and characterized [173].

Cotton fibers and PVA with H_2_SO_4_ yielded an acidic SPE tested in an EDLC device, showing 106% of the initial capacitance after 10,000 cycles [174]. Hydrolyzed cellulose and PVA were combined with Li_2_SO_4_ into an SPE tested in an EDLC device [175]. The beneficial effect of added Al_2_(SO_4_)_3_ is hard to follow, given the rather incomplete report. Apparently, it improves capacitance retention to 91% after 20,000 cycles. Cotton partially depolymerized with cellulase and subsequently combined with PVA yielded a flexible membrane, soaking in an aqueous solution of KOH yielded a GPE used in an EDLC device showing about 37% capacitance retention after 10,000 cycles in the optimum composition of the GPE [176]. Biopolymers synthesized with the help of bacteria [177] and algal-based polysaccharides [178] studied as constituents of SPE have been reviewed.

A GPE based on a cross-linked soybean protein isolate soaked with a neutral aqueous solution of Li_2_SO_4_ was used in an EDLC device, showing about 100% capacitance retention after 5000 cycles [179]. Soybean protein isolate grafted with polyacrylic acid for improved electrochemical performance and soaked in an aqueous solution of Li_2_SO_4_ was used in an EDLC device showing 87% of its initial capacitance after 5000 cycles [180]. Incorporation of lignin into this GPE yielded an electrolyte highly conductive even at *T* = −20 °C [181]. An EDLC device with this GPE kept 73% of its initial capacitance after 10,000 cycles. Chemical modification of soy protein isolate with acrylamide simplified its handling in preparing an SPE by soaking the obtained membrane in an aqueous solution of Li_2_SO_4_, which in turn enabled the assembly of an EDLC device, keeping 95% of its initial capacitance after 8000 cycles [182]. Cross-linked soybean protein isolate and hydroxyethyl cellulose with Li_2_SO_4_ as an ion source has been tested in an EDLC device keeping a slightly fluctuating capacitance^8^ along 5000 cycles [183]. A GPE based on tamarind seed polysaccharide with ammonium formate as an ion source has been prepared, characterized, and suggested for use in a supercapacitor [184]. An SPE prepared with polysaccharides derived from Chia seeds with Na_2_SO_4_ has been used in an EDLC device that kept 94% of its initial capacitance after 10,000 cycles [185].

Using seaweed-based alginate transformed into lithium alginate combined with lithium acetate yielded a flame-retardant GPE with 99% capacitance retention after 8000 cycles [186]. Membranes prepared from alginate with NH_4_Br as an ion conductor have been suggested for use in supercapacitors but have not been tested [187]. Lithium alginate cross-linked with lithium acrylate and vinyl silica nanoparticles formed an SPE of unknown stability [188]. A blend of sodium alginate and pectin dissolved in water was used to obtain a membrane suggested as SPE for supercapacitors [189].

Sodium alginate and PVA with added graphene oxide yielded an SPE tested in an EDLC device showing 96% capacitance retention after 5000 cycles (provided the errors in the report have been interpreted and corrected properly) [190]^9^.

Pectin and poly (ethylene glycol) were cross-linked using added CaCl_2_, which also acts as an ion source yielding an SPE assembled into EDLC devices and containing electrodes with pectin only or with the cross-linked material in the SPE [191]. In the fairly confused report, observed capacitance retentions of 77 and 83% after 5000 cycles cannot be attributed properly.

A GPE based on egg white adsorbed into an eggshell membrane with NaCl as an ion source has been tested in an EDLC device, providing 94% capacitance retention after 6000 cycles [192]. A similar approach has been tried again, yielding a device with 86% capacitance retention after 6500 cycles [193]. A SPE of lignin and a DEC was tested in a hybrid supercapacitor, showing 80% capacitance retention after 2000 cycles [194]. In a dye-sensitized solar cell (DSSC) integrated with a supercapacitor, an iodine-doped cellulose acetate propionate biopolymer was employed as an electrolyte for both the DSSC and the SC [195].

### 3.2. Plasticized^10^ Polymer and Gel^11^ Polymer Electrolytes in Supercapacitors

To further improve the properties of SPE, plasticizers can be added [26]. The result may be close to a gel electrolyte, although the approach starts from a different end: In the case of a gel electrolyte, a liquid is “solidified” or “gelled” by adding a gelling agent (see [2] for examples, more in this section below and in texts on gelling in the food industry) whereas a plasticizer is added to a solid (hard) polymer) yielding a more or less soft “gelled” or “gel-like” substance. The resulting material is sometimes called a gel electrolyte; according to the author’s specified preferences, results and reports can be found either in this section or in one of the following sections. When both terms (i.e., plastification and gel electrolyte) are used in one report (for an example, see [196]) the assignment follows the more prominently displayed term in, e.g., the title of the report. This terminological (or linguistic) uncertainty results in many variations in the description of the obtained electrolyte material, which also includes the entrapment of a liquid component.

PEO plasticized with ethylene carbonate with added LiTf^12^ and further ingredients have been examined with respect to possible use in energy storage devices [197]. A blend of PEO and PVP with (NH_4_)_2_Ce(NO_3_)_6_ as an ion source has been proposed as SPE [198].

#### 3.2.1. PVDF and PVDF-HFP

Polymers like PVDF, itself an insulator and more popular as a binder for electrode materials and as a material for porous separators in lithium ion batteries, and its copolymers can be dissolved and mixed with, e.g., ILs to yield solid electrolytes after evaporation of the solvent [199].

**Figure 8:**
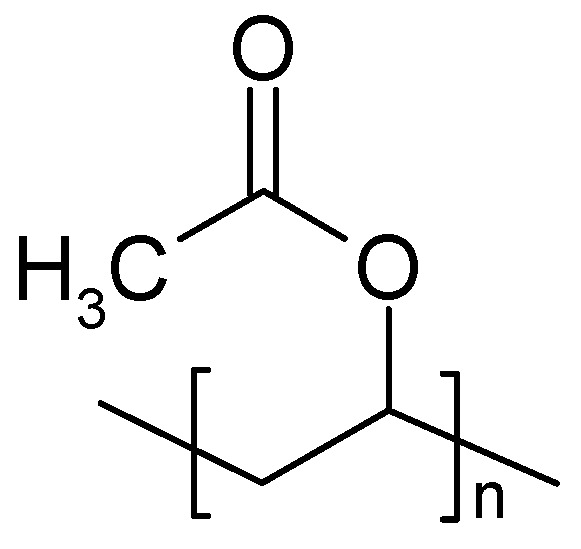
Polyvinylacetate

To a blend of PVDF and polyvinylacetate (Figure 8) dissolved in acetone, various amounts of an IL were added during the preparation of a SPE membrane [200]. An EDLC device prepared with an SPE of optimized composition kept about 90% of its initial capacitance after 5000 cycles.

An IL 1-ethyl-3-methylimidazolium tetracyanoborate (EMImTCB) was immobilized in poly (vinylidene fluoride-co-hexafluoropropylene) (PVDF-HFP^13^) yielding a plasticized or gelled polymer electrolyte [205]. The reported thermal stability is certainly due to the fact that the boiling points of ILs are typically much higher than those of organic solvents used for plastification elsewhere. A similar argument can be applied to the claimed wide window of electrochemical stability (the value reported versus a silver electrode in [205] remains mysterious—such a window does not need a reference electrode; it is an absolute value). Moreover, 3.8 V is not unusual for a nonaqueous electrolyte solution. A very similar approach has been tried with this copolymer and ionic liquids with 1,3-dialkyl-1,2,3-benzotriazolium as a cation and various anions yielding an electrolyte for an EDLC device [206]. Displayed GCD plots show a highly irregular behavior that is certainly not typical of an EDLC device, as claimed by the authors; stability was not examined. Using dibutyl 1,2,3-benzotriazolium tetrafluoroborate instead in the same approach, an SPE was prepared and used in a redox supercapacitor with PANI doped with this salt (the report states that the salt was doped with PANI–a highly unusual proposition) showing 89% capacitance retention after 3000 cycles [207]. With another IL, an EDLC device showed 18% capacitance decay after 10,000 cycles [208]. In a similar study, 25% capacitance loss after 2000 cycles was found [209] since a further example of this type with polypyrrole electrode stability data was not reported [210]. With this PVDF-HFP-IL combination as SPE in a redox supercapacitor, 90% of the initial capacitance was left after 6000 cycles [211]. A PVDF-HFP-IL SPE was used in an EDLC device, keeping 86% of its initial capacitance after 10,000 cycles [212]. A PVDF-HFP-IL SPE was tested in a redox supercapacitor, keeping 55% of its initial capacitance after 10,000 cycles [213]. Again, an SPE prepared by dissolving PVDF-HFP and an IL in acetone followed by solvent evaporation was tested in an EDLC device, showing 90% capacitance retention after 5000 cycles [214]. In a further example along these lines, an EDLC device was prepared and tested without reporting stability data [215]. Another similar example showed capacitance retention better than 96% after 10,000 cycles [216]. A redox supercapacitor with pure MnO_2_ as an active material and an SPE, as described in the previous examples, has been tested; data on stability have not been reported [217]. For a further example without stability data, see [218].

PVDF-HFP dissolved in acetone was mixed with an electrolyte solution of an IL with NaTf; after solvent evaporation, an SPE was obtained [219]. The assembled EDLC device kept 57% of its initial capacitance after 10,000 cycles. Using this SPE, the authors assembled a redox supercapacitor with ruthenium oxide- poly (3-methyl thiophene) electrodes with 66% capacitance retention after 5000 cycles [220]. Following this recipe for the SPE again, except for Mg(Tf)_2_ as an ion source, an SPE was prepared and tested in an EDLC device, which showed a 28% capacitance loss after 10,000 cycles [17]. An SPE based on PVDF-HFP with 1-ethyl-3-methylimidazolium bromide, propylene carbonate as a plasticizer, and Mg(ClO_4_)_2_ has been characterized [204]. An SPE obtained from PVDF-HFP dissolved together with NH_4_Tf and an IL in acetone without a plasticizer has been used in an EDLC device, showing 20% capacitance fading after 6200 cycles [221]. Further examples of PVDF-HFP combined with Ils have been reported [222].

PVDF-HFP plasticized with propylene and ethylene carbonate with added TEABF_4_ as ion sources have been studied as SPE for a redox supercapacitor, showing poor stability [223,224]. Elsewhere, as ion source NaClO_4_ was used, the SPE was tested in an EDLC device, showing 17% capacity fading after 10,000 cycles [225]. With NaTSFI instead, an SPE was used in an EDLC device, showing 25% capacity fading after 15,000 cycles with plain activated carbon electrodes; with composite electrodes, fading dropped to 7% [226].

PVDF-HFP plasticized with succinonitrile and with an added IL has been used as SPE^14^ in a hybrid supercapacitor showing about 80% capacitance retention after 2000 cycles [227]. The rather erratic changes in capacitance and the poor retention were attributed to a high internal resistance of the device, although the electrodes had been soaked with the electrolyte mix before assembly with a glass fabric separator. PVDF-HFP mixed with an IL and “plastic crystalline succinonitrile” was used as SPE in an EDLC device, which kept 80% of its initial capacitance after 10,000 cycles [228].

PVDF-HFP dissolved in acetone was mixed with a solution of LiTFSI in suberonitrile (1,6-dicyano-hexane, also acting as plasticizer), yielded an SPE after solvent evaporation [229]. An assembled EDLC device provided 90% capacitance retention after 20,000 cycles. To a solution of PVDF-HFP in acetone, a solution of LiTFSI in an IL was added, and the obtained film was used as SPE in an EDLC device with 90% capacity retention after 25,000 cycles [230]. These authors employed the same electrolyte but only different carbon electrode materials; the supercapacitor lost about 8% of the initial capacitance in the initial 2000 cycles [231]. A cell with the liquid electrolyte solution only lost about 70%.

A SPE prepared with minor difference by mixing a solution of PVDF-HFP in acetone with a solution of NH_4_Tf in an IL followed by solvent evaporation was used in supercapacitors with plain PEDOT:PSS and PEDOT:PSS/graphene nanoplatelet composite electrodes [232]. With the latter material, a higher specific capacitance was found; both systems lost about 10% of the initial capacitance after 2000 cycles.

For use in a lithium-ion capacitor, the copolymer just mentioned was treated with an electrolyte solution of LiClO_4_ in a mixed carbonate solvent, yielding, according to the author’s claim, a gel electrolyte [233]. Moreover, 86% of the initial capacitance was kept after 2400 cycles. A solution of PVDF-HFP in acetone was mixed with a solution of LiClO_4_ in a mixed carbonate solvent, and after solvent evaporation an SPE was obtained and tested in an EDLC device with no stability data reported [234]. Said copolymer (and not PVDF alone as claimed in the abstract of the report) was dissolved in acetonitrile; this solution was mixed with an electrolyte solution of TEABF_4_ in a mixture of propylene and ethylene carbonate also used as a plasticizer, subsequently yielding a GPE [235]. Combined with porous microelectrodes, a device with 86% capacitance retention after 5000 cycles was obtained. How penetration of the electrolyte into the porous electrodes may have been achieved remains unknown; device assembly was not described. Elsewhere, the said polymer was prepared as a porous membrane, which was subsequently soaked in an ionic liquid-base electrolyte solution [236]. An EDLC device prepared with this electrolyte kept a stable capacitance during 10,000 cycles. The formation of a gel or gel electrolyte was not specifically claimed. A redox capacitor with polythiophene electrodes and a microporous PVDF-HFP film soaked with a solution of LiPF_6_ dissolved in a mixture of propylene and ethylene carbonate as a “solid electrolyte” kept 97% of the initial capacitance after 1000 cycles [237]. PVDF-HFP combined with an IL was tested as an SPE [238]. The effect of lithium salt addition to an SPE of PVDF-HFP with an IL has been studied [239]. In an EDLC cell with optimized concentration, 80% capacitance retention after 1000 cycles was noticed.

PVDF-HFP mixed with Mg(ClO_4_)_2_ was dissolved in tetrahydrofuran, and after the evaporation of the solvent, a flexible film was obtained; how the propylene carbonate, which was also mentioned, was used remains unclear [240]. A redox supercapacitor with polypyrrole electrodes with unknown stability was tested. This SPE was used in a similar report by these authors elsewhere [202] for a supercapacitor with 50% capacitance retention after 5000 cycles, according to a displayed figure and 95% retention after this cycle number claimed in the text. This electrolyte was also used in a study of an EDLC device with silver-decorated carbon electrodes [241], with multiwalled carbon nanotubes instead of 96% capacitance retention observed after 5000 cycles [242]. It was again used in a symmetric redox supercapacitor, providing 91% capacitance retention after 5000 cycles [243]. PVDF-HFP again mixed with Mg(ClO_4_)_2_ and dissolved in tetrahydrofuran, but now plasticized with propylene carbonate, yielded a flexible polymer electrolyte sheet used in an EDLC device [244]. Modification of the employed carbon material with copper nanoparticles did not cause any visible change in CVs and GCD curves. Moreover, 91% of the initial capacitance was still present after 10,000 cycles. PVDF-HFP dissolved in acetonitrile mixed with Mg(ClO_4_)_2_ in propylene carbonate yielded an SPE after the addition of an IL [245]; test results other than stability have been reported elsewhere [246]. To the said combination with propylene carbonate as a plasticizer, fumed silica was added for improved ionic conductivity [247]. In the slightly confused report (the newly coined term “nanogel” is nowhere explained or justified), capacitance retention of the assembled EDLC device of 78% after 2300 cycles was found.

PVDF-HFP plasticized with ethylene carbonate with added GO for improved properties and with LiClO_4_ as an ion source has been tested in an EDLC device, stability has not been examined [248]. In a similar study, the copolymer was dissolved in acetone, zinc trifluoromethanesulfonate and the IL 1-ethyl-3-methylimidazolium trifluoromethanesulfonate were added, and the SPE finally obtained was used in a symmetric redox supercapacitor with polypyrrole electrodes [249]. Moreover, 26% of the initial capacitance was left after 500 cycles.

An SPE of PVDF-HFP with an IL and ceramic filler has been prepared and tested in an EDLC device [250]. To PVDF-HFP dissolved in acetone, an IL and various amounts of calcite (presumably obtained from blue mussel shells as reported by one of the authors elsewhere [251]) were added for improved mechanical stability and conductivity to obtain an SPE tested in EDLC devices [252]. Under optimum conditions, an 18% capacitance drop after 5000 cycles was observed.

To a solution of PVDF-HFP NH_4_F as an ion source and Al_2_O_3_ as a filler and to increase ionic conductivity were added, the obtained SPE was characterized and suggested for use in, e.g., supercapacitors [253].

PVDF-HFP dissolved in acetone and mixed with the ionic liquid 1-butyl-3- methylimidazolium tetrafluoroborate was spread over electrodes made of poly (3,4-ethylenedioxythiophene) yielding after evaporation of the solvent and assembly a redox supercapacitor [254]. No separator was needed; the dissolved electrolyte could penetrate into the active mass, providing an extended electrochemical interface. After an initial decrease in capacitance in the initial cycles, the capacitance was almost stable for 10,000 cycles. The authors reported a similar approach again with the same copolymer but a different IL 1-ethyl-3-methylimidazolium tris(pentafluoroethyl) trifluorophosphate aimed at an EDLC device with MWCNT electrodes [255]. In the initial 1000 cycles, about 20% of the capacitance was lost; up to cycle 10,000, the attained value stayed constant. PVDF-HFP^15^ dissolved together with EMIMBF_4_ yielded a solid electrolyte [256]. According to the report, the dry electrodes made from a graphene nanocomposite were pressed to these films without soaking the dry electrode with any liquid; whether the term iongel in one figure provides an explanation remains open. After 10,000 cycles, 85% of the initial capacitance was left. Further studies using this SPE and the corresponding preparation procedures have been reported [257]. A SPE based on PVDF-HFP with an added IL has been modified with graphene nanosheets for enhanced ionic conductance [258]. The assembled EDLC device kept 80% of its initial capacitance after 2000 cycles. The same material with another IL in an EDLC device kept 89% of the initial capacitance after 10,000 cycles [259]. An SPE based on PVDF-HFP with an added IL has been modified with graphene oxide and used in an EDLC device, which kept 80% of its initial capacitance after 5000 cycles [260].

PVDF-HFP dissolved in tetrahydrofuran was mixed with a solution of LiTFSI in a mixed carbonate solvent and yielded a GPE after evaporation of THF [261]. The assembled hybrid supercapacitor kept 95% of the initial capacitance after 3500 cycles. Similar examples have been reported elsewhere [262]. To PVDF-HFP dissolved in NMP, an IL and LiTFSI were added, yielding a SPE for a hybrid supercapacitor [263]. Moreover, 83% of the initial capacitance was retained after 4000 cycles. PVDF-HFP was dissolved in DMF with some added graphene oxide for improved mechanical strength, and an IL was added; a flexible GPE suitable for a 4.5 V cell voltage was obtained [264]. In a hybrid supercapacitor, up to 75% of the initial capacitance was still present. In a very similar approach, GO was added for improved ionic conductivity [265]. The EDLC device prepared with this SPE kept about 64% of the initial capacitance after 5000 cycles. Further examples of SPEs based on this copolymer and other Ils have been studied but not applied in supercapacitors [266].

An SPE based on PVDF-HFP with Zn(Tf)_2_, an IL, and DMF has been proposed for a zinc-ion capacitor [267]. The mixture was soaked into the positive porous carbonaceous electrode; the zinc electrode was simply pressed onto this without a separator. The device kept 88% of its initial capacitance after 10,000 cycles.

An SPE based on PVDF-HFP (and certainly not on an IL as claimed by the authors, although adding 300 wt.% suggests an unusual composition) with sodium thiocyanate^16^ has been suggested as a “futuristic approach” (!) for supercapacitor application [268]. A mixture of PVDF-HFP and zinc acetate was dissolved in DMF; the obtained SPE was tested in an EDLC device, and stability data were not reported [269].

A simple mixture of PVDF-HFP and fumed silica dissolved in acetone was used as SPE after solvent evaporation [270]. Elsewhere in the report, an electrolyte of Et_4_NBF_4_ in acetonitrile is mentioned when this SPE membrane was used in a redox capacitor with PANI as active mass and 70% capacitance retention after 5000 cycles. In a repetition of this work, 76% retention was found after 5000 cycles [271].

A mixture of PVDF-HFP and an IL dissolved in an acetone/DMF mixture was used for electrospinning to obtain an SPE^17^ [201].

The SPE options starting with PVDF-HFP dissolved in, e.g., acetone

- PVDF-HFP and IL
- PVDF-HFP and IL and electrolyte salt
- PVDF-HFP and IL and salt and plasticizer

have been compared [272]. With all combinations, severe capacitance losses were observed during 4000 cycles. The last combination provided the highest specific capacitance and 56% retention.

A similar copolymer of PVDF and trifluoroethylene dissolved in DMF was used in an EDLC device [273]. Apparently, the cell was assembled with the electrolyte, which is still rather liquid, a salt was not added, and stability was not examined.

PVDF-HFP was cross-linked with PAN, yielding a membrane that was soaked in a solution of acetonitrile and MeEt_3_NB_4_ and used in an EDLC device with 98% capacitance retention after 50,000 cycles [274].

To a solution of PVDF-HFP in acetone, mixtures of various Ils with succinonitrile were added, and after solvent evaporation, an SPE was obtained [275]. Performance in a supercapacitor was not examined.

Solutions of PVDF-HFP, PMMA, and NaSCN with various compositions were prepared to obtain an SPE membrane [203]. An EDLC device prepared with an SPE of optimized composition showed about 10% capacitance loss already after 600 cycles.

PVDF-HFP combined with tetraethyl ammonium tetrafluoroborate yielded a transparent gel used as an electrolyte in a self-charging supercapacitor with piezopolymer-containing electrodes for harvesting mechanical energy [276]. To PVDF-HFP dissolved in DMF, an IL EMITf and Al(Tf)_3_ were added to yield an SPE for an EDLC device showing 60% capacitance retention after 50,000 cycles [277]. When preparing a flexible device, the carbonaceous electrodes were soaked with the still liquid electrolyte, whereas for a coin-cell type device, this highly useful step (see Section 3.7) apparently was omitted for unknown reasons.

PVDF-HFP combined with poly (ethylene glycol) methyl ether methacrylate and trimethylolpropane ethoxylate triacrylate yielded after radical polymerization a semi-interpenetrating network was subsequently soaked with a solution of lithium hexafluorophosphate in ethylene carbonate/dimethyl carbonate to form a GPE [278].

A hybrid zinc-ion capacitor with an SPE of PVDF-HFP initially dissolved in acetone (the description is rather disjointed) combined with NH_4_TF in EMIMTF showed 20% capacitance fading after 100 (!) cycles [21].

#### 3.2.2. PEO

PEO plasticized with propylene/ethylene carbonate and nanoclay, with tetraethyl ammonium tetrafluoroborate as an ion source, has been used as a solid electrolyte in a hybrid supercapacitor with poly (3-methylthiophene) as positive and activated carbon as negative electrode [98]. About 30% of the initial capacitance was lost in 1000 cycles. PEO plasticized with aqueous KOH was also found to be compatible with an asymmetric supercapacitor with two different redox-active materials in the positive and the negative electrode with 97% capacitance retention after 10,000 cycles [279]. The same device showed poorer performance when a PVA-based or plain aqueous KOH solution (see below) was used. A cross-linkable poly (ethylene oxide)-poly (propylene oxide)-poly (ethylene oxide) triblock copolymer shows high IL electrolyte solution uptake [112]. An EDLC device kept 95% of its initial capacitance after 10,000 cycles. Sometimes the number of ingredients combined with PEO without providing any rational reason leaves open the question of the function of the additives and the proper assignment of the obtained electrolyte to any of the classes discussed in the present report; for an example, see, e.g., [128].

#### 3.2.3. PAN

**Figure 9:**
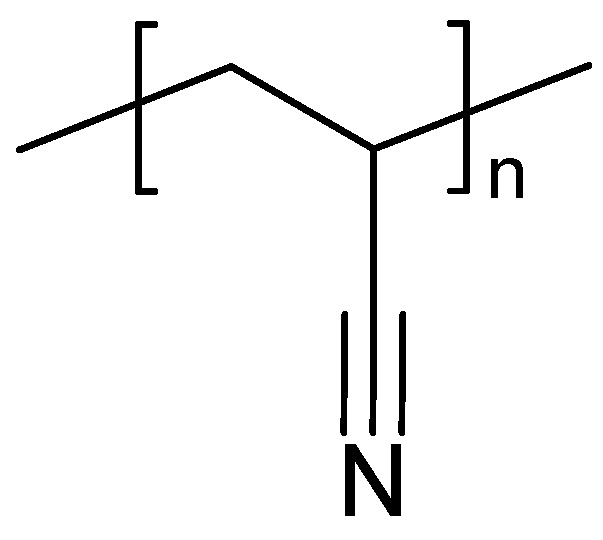
Polyacrylonitrile.

An example of a solid electrolyte of PAN (Figure 9) plasticized with ethylene and/or propylene carbonate has been reported; the material was designated a plasticized gel polymer electrolyte (GPE), supporting the concerns noted above [280]. A SPE based on PAN has been prepared by making a suspension of PAN and LiClO_4_ in propylene carbonate for a redox supercapacitor with PANI as active masses and 90% capacitance retention after 1000 cycles [281].

In a comparison of the three electrolyte systems, as aqueous and nonaqueous solutions, and as a GPE poly (acrylonitrile-polyhedral oligomeric silsesquioxane) in a redox supercapacitor, with a negative activated carbon electrode and nanoribbons of Co_3_O_4_ as positive electrodes, the cell with the GPE performed best [282]. Stability was not reported. Research progress of polyhedral oligomeric silsesquioxane as electrolyte material has been reviewed [283].

A solid electrolyte compatible with a redox-active electrode material based on PAN soaked (gelled) with an electrolyte solution of a mixture of ethylene and propylene carbonate and LiPF_6_ has been studied [127]. This is because the structural flexibility of the molecular chains is essential for the conduction crystallization of the SPE, which should be avoided. This can be supported by making copolymers or polymer blends. Typical conductivity values around 10^−3^ S·cm^−2^ have been collected [26]. Adding further materials, in particular nanoparticular inorganic ones like TiO_2_, as fillers can further enhance conductivity. The handling of materials has been reviewed [26]. PAN mixed with sodium polystyrenesulfonate has been used as an SPE in an EDLC device [284]. The properties of supercapacitors prepared with various polymers in gelled form have been compared [285]. The highest ionic conductivity was found with a PAN-based electrolyte, and the lowest was found with a PMMA-based one. The supercapacitor prepared with the latter electrolyte turned out to be more stable in terms of capacitance retention. As a copolymer of polythyleneglycol (PEG) and PAN, with dimethylformamide as a plasticizer and LiClO_4_, a solid electrolyte suitable for roll-to-roll manufacturing of an EDLC device with only a little capacitance decay after 30,000 cycles has been developed [286]. Poly (ethylene glycol diacrylate) combined with further components yielded an SPE for microsupercapacitors with 94% capacitance retention after 48,000 cycles [19].

PEG alone was used to immobilize acetonitrile in supercapacitors [287].

#### 3.2.4. PVA

**Figure 10:**
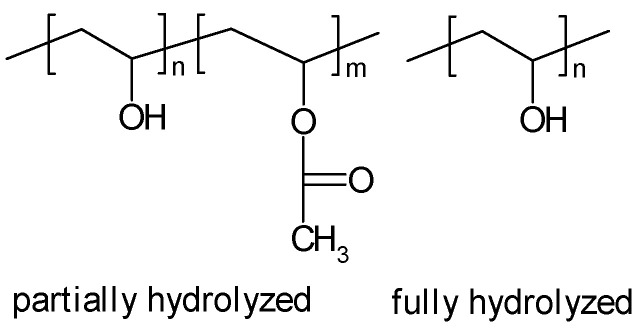
Polyvinylalcohol.

Polyvinylalcohol (PVA, see Figure 10) can be dissolved in hot water; upon the addition of, e.g., a solution of KOH, a gel is formed; depending upon water content, the product may also be called a gel-like solution [288]. Structural studies of PVA-based gels with small-angle X-ray scattering have been reported [289]. In addition, structural aspect dynamics of K^+^-ions in a PVA-SPE, with added KSCN, have been studied [290]. Electrochemical impedance studies of EDLC devices with a PVA-KOH SPE with associated modeling have been reported [291].

Further applications of PVA-KOH electrolytes have been reported [292,293,294,295,296,297,298,299,300,301,302,303]. In an asymmetric device with a negative electrode of activated carbon nanotubes and polypyrrole-coated Co(OH)_2_, an 89% capacitance retention after 5000 cycles was achieved [304]. Although a report on the use of PVA in an EDLC device initially left the impression that PVA is the only electrolyte constituent with added TiO_2_ nanoparticles as a filler and presumably an enhancer of mechanical strength [5]. A closer inspection reveals that KOH was added in the preparation procedure, yielding an electrolyte membrane that was attached to the carbonaceous electrodes by hot pressing. Stability was not examined, and evidence of the noticed porosity was not provided. PVA was combined with κ-carrageenan and KOH as an ion source and cross-linking agent and used as an SPE in an EDLC device with 95% capacitance retention after 2000 cycles [305]. In a lengthy and sometimes incoherent report on an asymmetric redox supercapacitor, the use of a PVA-KOH SPE is sometimes claimed; elsewhere, in the abstract and conclusions, a PVA-DMSO-EMIM-BF_4_ SPE that is not further specified is claimed [306].

The combination of PVA and H_2_SO_4_ has been employed in a typical example of an EDLC device using *N*-doped (by thermal treatment with added melamine) activated carbon derived from palm flowers [307]. The best-performing cell having carbon electrodes with intermediate nitrogen content provided 65% capacitance retention after 50,000 cycles. Reaction of the mixture of dissolved PVA and H_2_SO_4_ with glutaraldehyde yielded a SPE of significantly improved mechanical strength [308]^18^. In an earlier report, this combination was already employed with glutaraldehyde added as a cross-linking agent for improved mechanical stability [33]. The stability of the assembled EDLC device was not studied. An EDLC device was assembled with an electrolyte of PVA and H_3_PO_4_ with some KCl having a 91% capacitance retention after 3000 cycles [309].

An EDLC device with an SPE made only of PVA and H_3_PO_4_ was tested for 335,000 cycles without capacitance loss [310]. A solution of PVA and H_3_PO_4_ could be recrystallized by repeated freezing/unfreezing, yielding a porous membrane used as SPE in an EDLC device, which kept a stable capacitance during 10,000 cycles [311]. For further examples of this combination without specified crystallization see [312,313], other combinations of PVA with e.g., LiCl [314,315,316], LiClO_4_ [317,318], Li_2_SO_4_ [319], NaCl [320,321,322], Na_2_SO_4_ [13,323,324], KSCN [325], K_2_CO_3_ [326], H_3_PO_4_ [327,328,329,330,331,332,333,334,335,336,337,338,339,340,341], borates/boric acid [342], or H_2_SO_4_ [328,343,344,345,346,347,348,349,350] have been suggested and studied. The influence of the molecular weight of PVA and the concentration of KCl on the actual ionic conductivity of a GPE has been studied, and optimum parameters (i.e., a lower molecular weight is preferable) were communicated [351]. An EDLC device with such optimized GPE kept 88% of its initial capacitance after 5000 cycles. The influence of the added ion source on the performance of EDLC cells assembled with PVA-based SPEs has been studied with KCl, NaCl, and H_2_SO_4_ [352]. The highest capacitance was found with the acidic electrolyte. Unfortunately, with this electrolyte, the greatest capacitance loss of 15% after 1000 cycles was recorded; with KCl, the loss was 9%; and with NaCl, no loss was found. The influence of added acid concentration on observed supercapacitor capacitances has been studied [353]. In the case of sulfuric acid, an optimum was found with 2 M concentration; at 3 M concentration, leakage currents were very high. How a different nature of charge storage suggested in the report proceeds remains open. The effect(s) of an added surfactant to an SPE of PVA and ammonium acetate was studied by adding sodium dodecyl sulfate [354] at a concentration above the critical micelle concentration [355]. The improved performance of an EDLC device assembled with this SPE was claimed; stability was not examined.

PVA with Li_2_SO_4_ as GPE has been tested in an EDLC device, keeping 92% of its initial capacitance after 5000 cycles with optimum composition [356]. A PVA-based GPE with a concentration of LiCl high enough to form a Water-in-Salt system has been described [357]. The neutral pH enabled a rather high operating voltage (2.2 V); a single EDLC electrode combined with this electrode kept 84% of its initial capacitance after 20,000 cycles; results for a full cell were not reported. With lithium acetate as an ion source added to PVA, an SPE was formed for an EDC device, showing 90% capacitance retention after 8000 cycles. The addition of H_3_BO_3_ to an SPE of PVA and H_2_SO_4_ has been suggested without providing neither a clear reason nor an evident benefit [358]. PVA cross-linked with tannic acid, and H_3_PO_4_ added as an ion source yielded an SPE [359]. In a hybrid supercapacitor, 95% of the initial capacitance was still present after 1000 cycles.

In a study of PVA combined with H_2_SO_4_, a device with phase-change materials incorporated for thermal management was successfully examined [360]. The addition of h-BN nanosheets to a GPE of PVA combined with H_2_SO_4_ provided a major increase in ionic conductivity; an EDLC device assembled with this GPE showed 99% capacitance retention after 5000 cycles [361]. A PVA-H_2_SO_4_ gel electrolyte showing increased ionic conductivity after the addition of 1 wt.% of hydroxyethylcellulose in an EDLC device of unknown stability [362]. PVA-H_2_SO_4_ and PVA-H_3_PO_4_ without and with the addition of hydroxyethyl-cellulose have been compared using an EDLC device and vacuum infiltration of the electrolyte [363]. Electrochemical performance data were not reported.

A combination of PVA and H_3_PO_4_ suitable for inkjet printing has been developed and tested in a hybrid device, showing a stable capacitance during the initial 1000 cycles [364]. Various SPEs, including PVA and H_3_PO_4_, PEO plasticized with polyethylene glycol with added NaClO_4_, and PMMA plasticized with ethylene, propylene carbonate, and added NaClO_4_ have been compared with a redox supercapacitor using intrinsically conducting polymers as active masses [365]. As expected, a rather small operating voltage was noticed for a symmetric electrode combination, and stability data were not reported. A PVA and H_3_PO_4_ SPE have been prepared from standard materials; the function of diapers suggested as a source presumably of PVA in the title did not become clear in the somewhat confused description [366]. The specific capacitance values of assembled EDLC devices (with different PVA and H_3_PO_4_ ratios) depended greatly on the experimental methods, but without attracting the author’s attention, stability data were not reported.

A bilayer SPE based on PVA and LiCl was used to assemble a redox capacitor with low self-discharge [367,368,369]. For this purpose, the still liquid SPE coated on the positive electrolyte and soaked into it contained poly (sodium 4-styrenesulfonate) on the negative electrode poly (diallyldimethylammonium chloride). When assembled, the bilayer SPE significantly slowed down the ion movement associated with self-discharge. The device kept 60% of the initial capacitance after 2000 cycles. PVA combined with NaCl (this is presumably meant by food seasoning and table salt in the original report) has been tested in an EDLC device with graphene-based electrodes [370]. Moreover, 87% of the initial capacitance was left after 8000 cycles. An SPE based on PVA and NaCl with added glycerol was applied to an EDLC device by pouring the solution before solidification on the carbon electrodes (presumably for better penetration of the electrolyte into the electrode) yielding a device with 90% capacitance retention after 2500 cycles with a wide operating temperature range [371]. PVA with Li_2_SO_4_ and an IL was suggested as an “innovative” electrolyte for an EDLC device, showing 88% capacitance retention after 10,000 cycles [372]. The same combination has been studied elsewhere in an EDLC device with 90% capacitance retention after 3000 cycles [319]. A GPE prepared by adding an IL to a solution of PVA and ammonium acetate^19^ was used in an EDLC device with stability not examined [373]. An SPE of PVA with ammonium acetate as an ion source and an added IL was tested in an EDLC device, keeping 67% of its initial capacitance after 500 cycles [374]. In a highly confusing report on an EDLC device with an SPE of PVA with chitosan, sodium acetate as the ion source and glycerol as plasticizer has been tested in an EDLC device with almost constant capacitance during 500 (!) cycles [375]. The connection to the “MP issue” (presumably the occurrence of plastic microparticles in the environment) is nowhere addressed in the report beyond the abstract.

SPEs prepared from PVA^20^, ethylene carbonate, KI, and various Ils have been compared [376]. The highest capacitance was observed with 1-ethyl-3-methylimidazolium tetrafluoroborate; with this IL, 88% of the initial capacitance was retained after 3000 cycles.

A blend of PVA and chitosan plasticized with ethylene carbonate and lithium acetate as an ion source has been used in an EDLC device, showing around 20% capacitance loss after 1500 cycles [377]. PVA combined with phosphoric acid and an ionic liquid (1-ethyl-3-methylimidazolium tetrafluoroborate) has been used in a supercapacitor with activated carbon electrodes [378]. By treatment with nitric acid, additional surface functionalities have been created on the carbon. This added redox reactions as further storage mode to the EDLC-type charge storage. The redox reaction attributed to the ionic liquid is unknown and not specified. Capacitance retention was poor and got worse with the growing content of the ionic liquid (The reported numbers are mysterious).

PVA combined with magnesium triflate and an IL has been tested as SPE in an EDLC device, showing a slight increase of capacitance during 1000 cycles, possibly due to a decreasing internal resistance [379]. With a wider window of operating cell voltage (2 V instead of 0.85 V), retention decreased to 68 after the same number of cycles. These authors repeated this study using sodium triflate instead; again, a slight capacitance increase after 1000 cycles was observed [380].

A combination of PVA and an IL into an SPE showed 60% capacitance retention after 6000 cycles [381]. Such a combination has been studied with respect to relationships between composition, ionic conductivity, and mechanical properties; an SPE with optimum composition was tested in an EDLC device [382]. SPEs of PVA combined with various Ils have been examined with respect to possible use in printed supercapacitors [383]. A device with optimal combination kept 85% after 2000 cycles. To PVA with ammonium acetate as an ion source, various fractions of an IL were added, yielding an SPE tested in an EDLC device, which showed an 11% capacitance loss after only 250 cycles [40].

Elsewhere, instead of an IL, multiwalled carbon nanotubes (MWCNTs^21^) have been added to a mixture of PVA and NH_4_CH_3_COO, yielding an SPE suggested for use in supercapacitors [384].

An SPE from PVA with a deep eutectic solvent and hydroxylated boron nitride nanosheets was tested in an EDLC device showing 96% capacitance retention after 1500 cycles [385].

Combinations of PVA with heteropolyacids have been prepared and tested as solid electrolytes [386]. PVA mixed with phosphomolybdic acid has been used as an SPE in a hybrid supercapacitor of unknown stability [387]. PVA borate can be electrodeposited, immediately yielding good electrolyte/electrode contact. In addition, an increased cell voltage up to 2 V (and even higher) did not cause electrolyte decomposition [388]. This is because the possible higher cell voltage energy densities increased, too. Up to 89% capacitance retention after 5000 cycles is only slightly poorer than corresponding results with liquid electrolyte solutions.

**Figure 11:**
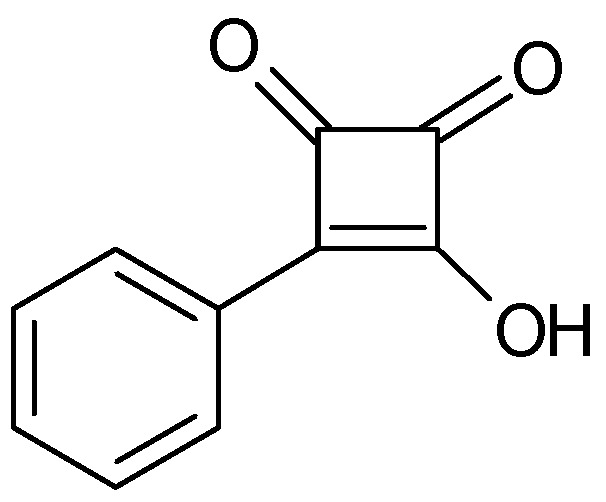
3-hydroxy-4-phenyl-3-cyclobutene-1,2-dione.

The relatively low ionic conductivity of PVA-based GPEs has been attributed to their high crystallinity. This can be disrupted by forming hydrogen bonds with, e.g., added agarose, a natural macromolecule [389]. Possibly, the addition of a plasticizer glycerol to a SPE of PVA and KSCN was aimed at the same result [325]. The respective EDLC device had fluctuating specific capacitance values along 380 cycles. To an electrolyte-improving electrode material utilization for an aqueous solution of PVA with some added sulfuric acid as a gelator 3-hydroxy-4-phenyl-3-cyclobutene-1,2-dione (Figure 11), which was also dissolved in an aqueous solution of sulfuric acid, was added; an SPE was obtained after solvent evaporation [390]. The assembled EDLC device showed a stable capacitance during 10,000 cycles; the performance was constant up to 250 μm electrode thickness.

A blend of PVA and sodium polyacrylate with KOH as an ion source has been used in a hybrid supercapacitor [391]. Specific capacitance, as well as capacitance retention during 1000 cycles, strongly depended on the mass ratio of the positive Ni(OH)_2_ and negative activated carbon electrode. Generally, a poor stability of the Ni(OH)_2_ electrode was noticed. Overviews of PVA-based SPEs are available [392,393].

#### 3.2.5. PMMA

A stretchable EDLC-supercapacitor prepared with an SPE from PMMA and an IL kept 88% of its initial capacitance after 1000 cycles [394]. The addition of KOH as an ion source to PMMA also increased its amorphicity, supporting conduction; an EDLC device assembled with this SPE was not examined for stability [395].

**Figure 12:**
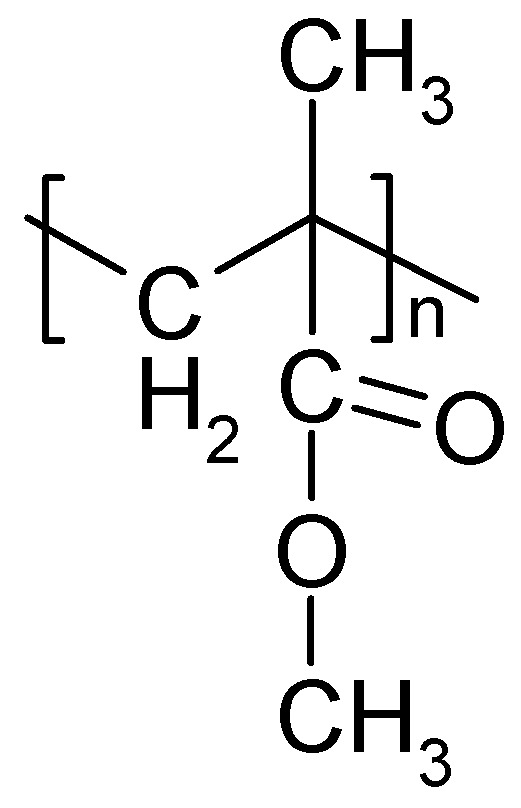
Poly (methyl methacrylate).

Solid electrolytes based on poly (methyl methacrylate) (Figure 12) profit from several advantages of this polymer, including simple synthesis, low density, mechanical stability, weak binding to ions of added electrolyte, and high charge carrier mobility; they suffer from low ionic conductivity. Application of plain PMMA is difficult because its brittleness prevents good contact with an electrode. Thus, various modifications of PMMA have been examined to remediate this flaw and to improve ionic conductivity; overviews are available [396,397]. Initial attempts, including copolymerization, the addition of plasticizers or organic fillers, and copolymerization, did not yield significant progress or failed entirely [398]. Combination with ionic liquids provides some moderate improvements. PMMA-based electrolytes have been reviewed [397]. To a solution of LiTFSI in adiponitrile and succinonitrile, poly (methyl methacrylate) was added; the obtained SPE was tested in a hybrid supercapacitor keeping 88% of its initial capacitance after 5000 cycles [399]. A SPE of poly (methyl methacrylate), tetrabutylammonium tetrafluoroborate, and acetonitrile has been tested in a redox supercapacitor with stability data provided only for single electrodes [400].

A SPE of poly (methyl methacrylate) grafted natural rubber with ammonium triflate as an ion source plasticized with ethylene carbonate has been tested in an EDLC device [401]. Stability data were not reported.

A polymer prepared from glycerylmonomethacrylate with phenylboronic acid has been prepared (why the product was called boron-doped remains unclear) and combined with DMF and LiClO_4_ into a gel polymer electrolyte [402]. An EDLC device with this electrolyte kept 90% of its initial capacitance after 3000 cycles. A SPE of photopolymerized glycidyl methacrylate dissolved in DMF with added LiClO_4_ as an ion source and hierarchical porous carbon microspheres added for wider voltage window and improved heat resistance was tested in a hybrid supercapacitor keeping 92% of its initial capacitance after 5000 cycles [403].

A related monomer 2-hydroxy-3-phenoxypropylacrylate was UV-light polymerized in the presence of propylene carbonate and LiClO_4_, yielding a gel polymer electrolyte [404]. Because polymerization was performed after soaking this solution into the porous activated carbon electrode body, a good interfacial electrode/electrolyte contact was established, but nevertheless, a separator was needed in device assembly. Moreover, 81% of the initial capacitance was retained after 9000 cycles, suggesting a stable interfacial contact.

Polyethylene glycol diacrylate combined with an ionic liquid EMIMTFSI and LiTFSI yielded a solid electrolyte named “ionic-gel polymer electrolyte” (IGPEs) used for an EDLC device with 86% capacitance retention after 10,000 cycles [405].

Poly (ethylene glycol) dimethacrylate was polymerized in a mixture with acetonitrile and an ionic liquid after it was soaked into a cellulose separator [406]. In an EDLC device, 20% of the initial capacitance was lost after 10,000 cycles.

An acrylate rubber not specified more closely soaked in a solution of tetraethylammonium tetrafluoroborate in acetonitrile served as an electrolyte in a redox supercapacitor with polyaniline electrodes keeping 88% of its initial capacitance after 10,000 cycles [407]. The electrodes were soaked with the electrolyte solution before assembly.

A solid electrolyte (ormolyte) has been prepared by a sol-gel process starting with tetraethoxy orthosilicate and tetraethylene glycol combined with various magnesium salts [408]. An EDLC device could be cycled more than 1000 times.

A flexible copolymer film of vinyl acetate and 1-ethyl-3-vinylimidazolium cations (a polycation) with the bromide anion serving as the main conductor and a vinyl chain as the molecular backbone and a wide electrode potential window of electrochemical stability has been prepared [136]. The prepared EDLC-type supercapacitor kept 90% of its initial capacitance after 5000 cycles. The performance was attributed in part to an electrolyte/electrode interface utilizing the flexibility of the electrolyte, which supports fast charge transfer. Polymer electrolytes, as widely employed in lithium-ion batteries, are ion-conducting polymeric materials solid at room temperature [409]. Because their ionic conductivity is relatively low, they are, at first glance, of little interest for supercapacitor application. Nevertheless, their flexibility, bendability, and stretchability, which depend on the polymer itself, and—when applicable—added further ingredients make them candidate materials worth further examination [410]. More examples of solid electrolytes, as applied in supercapacitors that are flexible, wearable, etc., can be found in [411]. The importance of flexible semi-solid or solid electrolytes has been highlighted in an overview [412]. Blends of PILTFSI with various ILs suggested as solid polymer electrolytes for EDLC-type devices have been compared [413]. Differences in terms of actual conductivity and electrochemical stability window were noticed and attributed to properties of the added IL.

A free-standing electrolyte film was prepared from a mixture of a partially fluorinated, microphase-separated comb copolymer of superhydrophobic poly (2,2,2-trifluoroethyl methacrylate) and amphiphilic crystalline poly (ethylene glycol) behenyl ether methacrylate with an ionic liquid [EMIM,TFSI] also acting as separator [125]. An EDLC-type supercapacitor outperformed a corresponding cell made with a PVA-based gel electrolyte. A similar approach with different starting materials has been reported [126]. These authors reported on a further copolymer poly(styrene-b-butadiene-b-styrene)-g-poly(ethylene glycol) behenyl ether dissolved in THF and mixed with an IL [414]. The mixture was cast on an EDLC-type electrode; two electrodes were assembled into a supercapacitor without a separator, showing 87% capacitance retention after 5000 cycles.

A flexible solid-state EDLC-type supercapacitor capable of withstanding elevated temperatures (120 °C) based on a solid electrolyte of a poly (aryl ether ketone)-poly (ethylene glycol) copolymer has been reported [117]. Negligible capacitance losses after 2000 cycles were found.

Polymer electrolytes inspected so far are mixtures of various solid and/or liquid materials; to date, no single-phase single material has apparently been studied successfully as an electrolyte for a supercapacitor. In a review of polymer blend nanocomposites as applied in energy storage devices, including supercapacitors, nanocomposites cannot be found for the latter application [46].

#### 3.2.6. PBI

**Figure 13:**
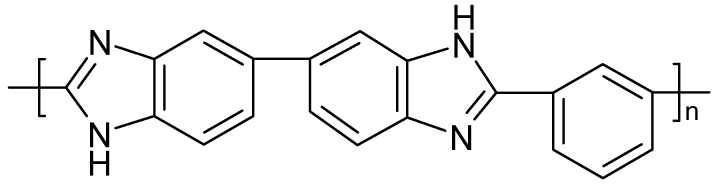
Polybenzimidazole.

Polybenzimidazole (PBI, see Figure 13) has been suggested as an SPE apparently without any further modification; just a thin film was found to be sufficient even without an additional separator [415]. Sufficient interfacial electrode/electrolyte contact was established by soaking the electrodes with a solution of PBI in dimethylacetamide before assembly. Only at the very end of the report was the soaking of the whole device with an aqueous solution of KOH mentioned! This SPE has been used in a study with a hybrid supercapacitor using a positive layered double hydroxide electrode and a negative activated carbon electrode, providing 93% capacitance retention after 10,000 cycles [416]. Films of PBI were coated onto carbonaceous electrodes; the assembled device was soaked in either aqueous KOH or H_3_PO_4_ [417]. Along 10,000 cycles with the former electrode, a capacitance loss of 7% was found; with the latter electrolyte, a growth of about 25% was noticed.

A porous film of PBI was soaked with an IL (1-(3-trimethoxysilylpropyl)-3-methylimidazolium chloride) [418]. The subsequent hydrolyzation of the latter resulted in the formation of an -O-Si-O network, improving the mechanical stability of the film and increasing water uptake and ionic conductivity. An all-solid-state EDLC supercapacitor kept 91% of its initial capacitance after 10,000 cycles.

A film of PBI soaked with an aqueous solution of KOH (the authors called this “doped” for unknown reasons) has been used as a solid electrolyte in an asymmetric supercapacitor combining an activated carbon negative and a Ni(OH)_2_ positive electrode [419]. Cycling stability and capacitance retention were found to be disappointing. Said “doping” was repeated by the same authors for a hybrid supercapacitor with layered double Ni-Co hydroxide, which showed a 95% capacitance retention after 5000 cycles [420]. PBI soaked with H_3_PO_4_ (the term appears to be somewhat misleading) has been used as SPE in an EDLC device, keeping 80% of its initial capacitance after 1000 cycles [421].

#### 3.2.7. PVP

**Figure 14:**
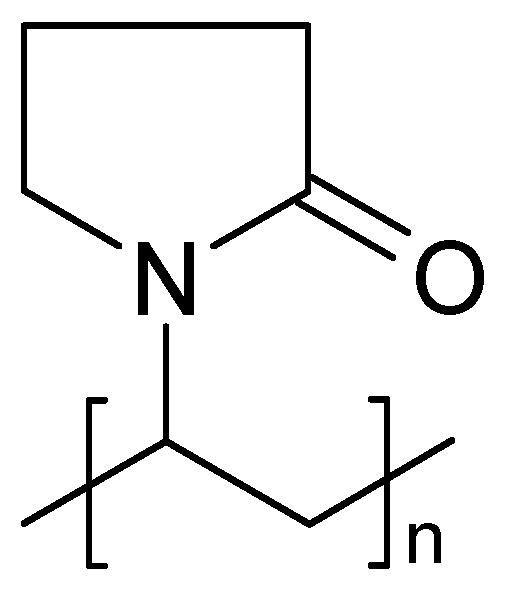
Polyvinylpyrrolidone.

Polyvinylpyrrolidone PVP (Figure 14) with NH_4_I as an ion source and ethylene carbonate as a plasticizer has been prepared and characterized. Its application as SPE in an EDLC device yielded an inconsistent cyclic voltammogram and some inconclusive impedance data [422]. The IL 1-ethyl-3-methylimidazolium hydrogen sulfate (EMIHSO_4_) was immobilized into a blend of PVA and PVP; the obtained film was used in an asymmetric supercapacitor showing stable capacitance for 1000 cycles [423].

The dual-use option of PVP as a binder and solid electrolyte (with added phosphoric acid) combined with its biodegradability as an environmental advantage has been highlighted in a study of an EDLC device using rGO obtained by recycling used graphite from expired batteries [424]. Although the recycled electrode material was claimed to be equivalent in terms of performance to freshly prepared rGO, the capacitance retention of 97% after only 2000 cycles leaves room for improvement for an EDLC device. Vinylpyrrolidone, polymerized in the presence of an IL, yielded an SPE, which was subsequently tested in an EDLC device with a stability that is not reported [398]. PVP was cross-linked with polyacrylamide and combined with H_3_PO_4_, yielding an SPE for an EDLC device showing 86% capacitance retention after 5000 cycles [425]. A blend of PVP and PVA with an optimized amount of added KI has been studied as SPE in a DSSC and a supercapacitor with unknown stability [426].

#### 3.2.8. Miscellaneous Synthetic Polymers

Poly (*N*-vinyl imidazole) deposited from its solution with KOH was tested as electrolyte for a microsupercapacitor [427]. From the highly fragmentary description, it can be deduced that an EDLC device with carbon electrodes was manufactured and tested, although the GCD curves suggest otherwise. Nothing is reported on stability; suddenly appearing claims about capacitance retention in the conclusions are contradictory.

A polyacrylamide-based hydrogel electrolyte sheet suitable for operation even at *T* = −35 °C was coated with polyaniline on both sides [428]. The supercapacitor thus created kept 86% of its initial capacitance after 5000 cycles. A PAM-based SPE with Li_2_SO_4_ as an ion source has been tested in an EDLC device with very small capacitance loss during 5000 cycles [429]. Improved electrode-electrolyte interaction was achieved by casting the electrolyte “precursor solution” onto the electrode. A hydrogel of polyacrylamide with clay as a cross-linker has been tested in an EDLC device, with capacitance retention not reported [430]. Polymerization of acrylamide in the presence of xanthan yielded a less crystalline and, thus, better ion-conducting solid; with added lithium acetate, an SPE for an EDLC device was obtained, which showed an 82% capacitance retention after 10,000 cycles [431].

A self-healing zwitterion-containing polyelectrolyte hydrogel has been prepared by copolymerization of acrylamide, zwitterionic sulfobetaine methacrylate, and 2-acrylamido-2-methyl-1-propanesulfonic acid (AMPS) has been used in an EDLC device with 97% capacitance retention after 1000 cycles [432]. A zwitterionic semi-interpenetrating polymeric hydrogel was prepared from sulfobetaine methacrylate and further constituents with NaClO_4_ as an ion source, yielding an SPE for an EDLC device with 98% capacitance retention after 5000 cycles [433].

#### 3.2.9. Biopolymers

Polymer electrolytes based on algae polysaccharides as promising alternatives to conventional synthetic materials have been reviewed [178]. A porous lignocellulose membrane prepared from natural raw material showed remarkable uptake of aqueous KOH solution when subsequently used as an electrolyte in an EDLC device with 92.5% capacitance retention after 10,000 cycles [160].

A blend of chitosan and dextran with wild honey as a plasticizer and a major weight fraction of NH_4_SCN as an ion source has been studied as SPE [434]. Rheological properties of GPEs based on chitin and ionic liquids have been reported [435]. SPEs based on chitin and an IL or cellulose, an IL and DMF have been prepared and tested (presumably, no details are provided) in an EDLC device, which kept about 80% of the initial capacitance after 10,000 cycles [436].

To an aqueous solution of dextran, NH_4_NO_3_ as an ion source and glycerol (presumably as a plasticizer if that was meant by the author’s term “Glycerolized”) were added, yielding a SPE tested in an EDLC cell, which kept its energy density during 1000 cycles with some fluctuations [437].

A betain-based zwitterionic polymer electrolyte with added betaine-functionalized graphene oxide particles has been prepared and tested in an EDLC device, which showed a stable capacitance during 5000 cycles [438].

#### 3.2.10. SPEs Based on Gelling Agents

Plasticized SPEs created by treating a polymer (whether it is considered as an SPE or just a polymer, perhaps even used as a binder for active masses in an electrode hardly matters) with a suitable liquid yielding a “gel-like” electrolyte have sometimes been called gelled polymer electrolytes, they have been presented above in this section. This has included polymeric materials, which are both present as membranes, sheets, or even powders (a detail that is minor but may contribute to the common confusion in this field). In reports about electrolytes for supercapacitors, materials are sometimes obtained by a different procedure starting from a liquid electrolyte or electrolyte solution, as already indicated above: A gelling agent is added to the liquid. In case the gelling agent is a polymer, the result will be treated below in this section; for other examples with different non-polymeric gelling agents, see [2].

In a typical example, agarose is used as a gelling agent with an aqueous solution of NaCl [439]. In a redox supercapacitor, an 80% capacitance retention after 1200 cycles was noticed. Cell assembly was not described, possibly the rather high liquid content in the GPE supported penetration of some electrolyte solution into the porous electrode body. A GPE based on agarose with lithium acetate as an ion source and added graphene oxide has been prepared [440]. An EDLC device with this GPE kept 91% of its initial capacitance after 4800 cycles.

The poor ionic conductivity of PAN can be improved significantly by gelling with organic solvents. A variety of mixtures of common organic solvents with LiClO_4_ as an electrolyte has been used for this purpose; results have been compared; performance data reached those of supercapacitors with liquid electrolyte solutions [196].

A bioinspired gel soaked with a highly concentrated solution of LiTFSI has been suggested as an electrolyte for an EDLC device also operating at temperatures down to *T* = −77 °C with 94% capacitance retention after 10,000 cycles [441]. Fitting wetting behavior was claimed to ensure penetration of the gel into the porous electrode body.

The role of GPEs has been studied in more detail; results reveal binder-like contributions toward device integrity and ion reservoir contributions—in particular, inside electrode pores [442]. The latter effect contributes to high rate capability.

A flame-resistant gel electrolyte prepared from chitosan, sodium borate, and levulinic acid provided 86% capacitance retention after 50,000 cycles in an EDLC device [443]. A self-healing GPE based on chitosan cross-linked with a further macromolecule has been tested in an EDLC device, showing 80% capacitance retention after 10,000 cycles [444].

With a gelatin-based (and not acetate-based as claimed in the title) SPE with glycerol as a plasticizer and sodium acetate as an ion source, an EDLC device showing 39% capacitance loss in 50,000 was assembled [445]. Fish skin-derived gelatin combined and used in a device somewhat mysteriously designated as “non-Faradaic-based” with 70% capacitance retention after 150 cycles [446]. Gelatin with added glycerol as plasticizer and NaCl as an ion source has been tested in an EDLC device, showing a stable capacitance along 12,000 cycles with electrodes with chitosan as a binder and only minor contributions to charge storage by redox reactions [447]. Chitosan-enforced gelatin yielded an SPE, which subsequently soaked in an aqueous solution of (NH_4_)_2_SO_4_ and NH_4_Cl, and was used in an EDLC device with 81% capacitance retention after 2000 cycles [448]. The SPE showed remarkable fire retardancy and low-temperature performance.

Glutaraldehyde-cross-linked corn starch with NaClO_4_ has been used as a GPE in an EDLC device, which showed stable capacitance along 100 (!) cycles [449]. Corn starch was used without crosslinking, but lithium acetate and TiO_2_ nanoparticles were added for reduced crystallization as GPE with unknown stability of a device assembled with it [450]. An SPE of corn starch with NaCl as an ion source and an added IL has been suggested for an EDLC device [451]. SPEs based on corn starch with an added IL have been studied in “dual energy devices” yielding rather incoherent results [452]. The influence of the anion of the IL added to a corn starch-based SPE with LiPF_6_ as an ion source has been examined; apparently, the ionic conductivity of the SPE that was finally observed was of interest [453]. Although hexfluorophopshate ions were introduced with LiPF_6_ in both cases, the EDLC device with a triflate-containing IL showed, somewhat confusingly, a much better stability of the recorded capacitance.

Cellulose nanofibers^22^ soaked with H_2_SO_4_ yielded an SPE, the prepared supercapacitors showed at best a capacitance retention of 75% after 5000 cycles [454].

A natural DNA-based SPE with LiCl as an ion source used in an EDLC device enabled capacitance retentions up to 94% after 200,000 cycles [455].

A comparison of the electrochemical performance of various supercapacitors with several GPEs/SPEs has been reported; in the selected cases, an SPE based on PEO/NaOH performed best [456].

### 3.3. Ion Exchange Polymers as Electrolytes in Supercapacitors

**Figure 15:**
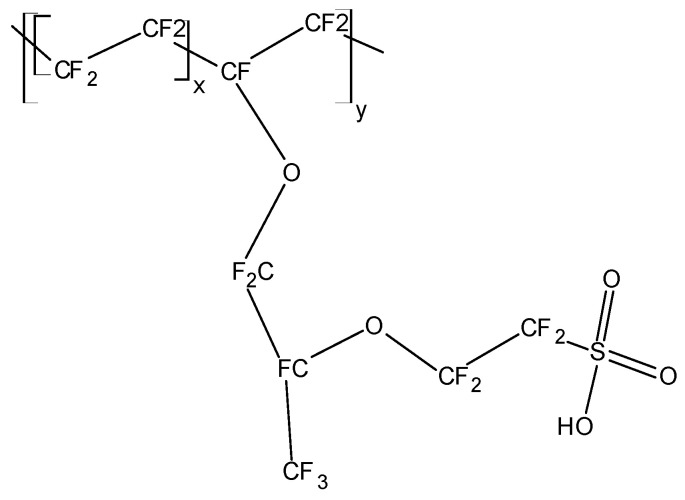
Molecular structure of sulfonated perfluorocarbon polymer polytetrafluoroethylene PTFE.

Attachment of ionogenic functional groups like carboxylic or sulfonic acid groups to polymeric materials not supporting ion movement themselves (these true ion conductors have been discussed above in Section 3.1) yielded ion-conducting polymers actually behaving like ion-exchange materials. These materials, frequently called SPE (a slightly unwelcome narrowing of the much broader term), have been quite successful in fuel cell and water electrolysis technology. A typical example, perfluorinated polyethylene (polytetrafluoroethylene PTFE), which was partially substituted with sulfonate ionogenic groups, is shown in Figure 15.

The sulfonic groups can release protons, providing ionic conduction; they stay in place and provide sites for moving protons to jump at and to jump off again [457]. This mechanism of conduction, which closely resembles the Grotthus transport mechanism responsible for the extra-high ionic conductivity [458], is just one option. A second one is the transport of protons using, e.g., water molecules as a vehicle (i.e., H_3_O^+^-species) in a diffusion-like manner requiring particular levels of humidity; a third option is proton transfer based on chain segment motion, which enables proton transfer. The use of ion conductor polymers, both cation- and anion-conducting, in supercapacitors, has been studied extensively [459]. A Nafion^®^-membrane was used in a supercapacitor with Keggin-type heteropolyacids and RuO_2_ as electrode materials [460]. The electrodes were wetted with a minimum amount of aqueous 5.3 M H_2_SO_4_ before they were pressed on both sides of the membrane. The stability of the capacitance along 80 (!) cycles was called excellent. Nafion^®^, as well as Aquivion^®^ CEMs, were used with electrodes soaked with an aqueous solution of 1 M Na_2_SO_4_ for improved electrode/electrolyte contact (see also Section 3.2) [461]. Application of an Aquivion^®^^23^ CEM in a hybrid supercapacitor with unknown stability has been reported [462]. These authors reported for this system in another study again no stability [463]. In a further study, the authors reported stable capacitance values along 10,000 cycles [464].

Various types of Nafion^®^ were tested in an EDLC device prepared by a simple lamination technique [465]. Stability was not examined. A rather mysterious organic electrolyte, designated as an “ionic polymer metal composite”, turned out on closer inspection to be a Nafion^®^ membrane cast from its solution, which was subsequently soaked in a platinum (!) salt solution; it was used with a carbonate-based electrolyte solution of LiPF6 in an EDLC device with unknown stability [466]. The incorporated platinum nanoparticles increased the ionic conductivity of the membrane, which actually may be called, more precisely, a separator. An SPE of solution-cast Nafion^®^ was used in an EDLC device showing only a few percent capacitance loss during 1000 cycles [467]. Using the Nafion^®^ solution (i.e., using Nafion^®^-functionalized carbonaceous material) as a binder in the electrode greatly helped achieve this performance, without which the capacitance decreased quickly with cycling. Spray-coating a dispersion of multiwalled carbon nanotubes in aqueous diluted sulfuric acid yielded an EDLC device [468]. Power density decreased by 12% after 2000 cycles, and further stability data were not reported.

The ion conductivity of various types of Nafion^®^ has been reported [469]; for a comparison of conductivity data of Nafion^®^ 117 and a structurally related CEM, see [470].

Overviews on the application of these SPEs in supercapacitors are available [22]. Depending on the identity of the ionogenic groups, the polymers will be cation-conductors with anionic groups attached to the polymer backbone and will behave like a cation-exchange polymer (CEM) or anion-conductors behaving like an anion-exchange polymer (AEM). The development of the former class of materials has moved much farther than that of the latter. Corresponding reviews covering selected topics are available; proton-conducting materials, in particular, have been treated in [457].

In addition to the high price, drawbacks of the materials related to the acidic character of this membrane have been known for a long time in fuel cell research. With respect to supercapacitors, interest in alkaline ion exchange membranes has resulted in achievements being reviewed together with those of alkaline (OH^−^-conducting) solid polymer electrolytes [49]. A presumably more common, most likely fundamental, problem is the establishment of a sufficiently close contact between a solid electrolyte and a solid, highly porous electrode. This contact is essential because it will only happen at these places of contact charge transfer (in the case of redox supercapacitors) and/or ion accumulation and dissipation (in the case of EDLC devices). With a smooth electrode (e.g., a lithium metal foil), this will not be a problem. Moreover, when a liquid electrolyte (solution) is used, the problem does not appear because the liquid will penetrate into the porous body. To overcome this problem of poor electrolyte/electrode contact Nafion^®^ ionomer was mixed with the RuO_2_ electrode material used as an active material for a redox capacitor. For a typical example with a commercial Nafion^®^-membrane as a solid electrolyte, see [471]. A similar approach with activated carbon as electrode material yielding an EDLC device has been described later [472,473,474]. Data on stability were not reported. According to the experimental details reported, this wetting step was not applied when preparing a hybrid device with a positive MnO_2_ electrode; nevertheless, only 5% capacitance loss after 10,000 cycles was reported [475]. With Nafion^®^-membrane ion-exchanged with Na_2_SO_4_ in such a device, the initial capacitance showed an increase of 6% after 2500 cycles [476].

The use of fluorinated organic compounds is meeting growing environmental concerns focused on various steps of their lifecycle—in particular, during synthesis and disposal when reaching the end of their useful lifetime. Thus, research efforts have been directed at replacements without fluorine. Further noticed flaws, in particular the need to keep these materials in a specific state of hydration needed for sufficiently fast ion movement, were another driving force. Most of the proposed alternatives start with a hydrocarbon backbone containing benzene and/or heteroaromatic repeat units with suitable substituents providing the required ionogenic functionalities. For an overview see [457].

### 3.4. Polymerized Ionic Liquids

Ionic liquids (IL) form a class of ionically conducting materials also of interest for use in supercapacitors [2]. At least those liquids at typical operating temperatures of a supercapacitor, i.e., room temperature ionic liquids RTIL, face the same problems and concerns valid for electrolyte solutions. Thus, ILs that can be polymerized have attracted attention as conceivable candidate materials. An early comparison of ionic conduction in such polymers and in mixtures of ILs, taking into account molecular details of the polymers and conceivable effect on conduction, has been reported [477]. Overviews for IL in GPEs are available [478,479,480], and further general aspects have been discussed [481,482]. The increase of ionic conductivity of SPEs by incorporation of Ils has been considered in [483].

**Figure 16:**
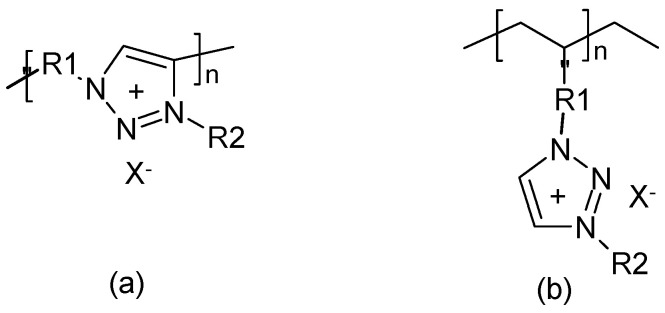
Structural options of PILs, (**a**): IL in polymer chain; (**b**): IL as a pendant group.

A classification of polyionic liquids (PILs) defined as a class of solid polyelectrolytes based on polymerized ionic liquid molecules into polycationic, polyanionic, and polyzwitterion ones has been proposed [484]. In the first group, polymers are based on cations of established ionic liquids with a correspondingly wide variation of encountered polymers. In the second group, there is less variation, as already known from ionic liquids with a much smaller number of anions. Moreover, 1,2,3-triazolium-based PILs (with a further acronym TPILs) have been examined more closely [484]. Figure 16 illustrates the basic options, with the IL-unit being part of the polymer chain (a) and the Il-unit attached to a polymer backbone (b). An overview of PILs is available [485]; the charge transport mechanism in PILs has been studied in detail [486].

A PIL (poly (1-vinyl-3-propylimidazolium) bis(fluorosulfonyl)imide) has been combined with an IL by dissolving the PIL in acetone first with subsequent addition of the selected IL yielding a SPE [487]. This solution was first dropped onto the carbonaceous electrodes of the intended EDLC device for better electrode/electrolyte contact. A capacitance retention of about 76% after 5000 cycles can be derived from a displayed figure not discussed in the report. A PIL, namely poly (diallyldimethylammonium) bis(trifluoromethanesulfonyl)imide, has been combined with several different ILs for a study of the influence of IL properties like ionic conductivity and electrochemical stability on EDLC device performance [413]. Several optimum combinations could be identified. The influence of ions in IL/PILs on device properties has been studied [488]. Self-healable PILs have been studied [489]. Radiation-induced copolymerization of an IL with octavinyl polyhedral oligomeric silsesquioxane followed by the addition of LiPF_6_, which yielded an SPE, has been reported [490]. The stability of the device formed therewith was not examined.

A copolymer of an IL with PMMA has been prepared and blended with PVDF-HFP and tried in an EDLC device showing 80% capacitance retention after 2000 cycles [491]. An overview of ILs in SPE is available [492]. Radiation-induced polymerization of a mixture of 1-vinyl-3-ethylimidazolium tetrafluoroborate and ethylene glycol diacrylate with added Ti_3_C_2_T_x_ yielded a GPE for an EDLC device with less capacitance retention (according to displayed data) than the 93% claimed in the text after 300 cycles [493]. A combination of pyrrolidinium-based PI and corresponding IL was used as SPE in an EDLC device with unknown stability [494].

A PIL containing an IL and modified with 2D silica nanoplates for enhanced ionic conduction was prepared [495].

### 3.5. Polymer Electrolytes with Added Redox Systems

For the added storage capability of supercapacitors, the addition of reversible redox systems to the electrolyte (solution) has been proposed and studied extensively [2]. For efficient utilization of the added charge carriers, they have to move toward the electrode and away from it freely; apparently, they do not recommend the use of solid polymers. On the other hand, excessive mobility of these charge carriers results in self-discharge [31], in the worst case, by some shuttle effect between the electrodes, which is slowed down by such polymer electrolyte. The use of redox materials incorporated into the electrodes, e.g., as a composite, is somehow related, which depends on the solubility of the added redox material. In the case of completely insoluble materials, the electrode is just a redox electrode. Some examples will be presented below to illustrate the options and highlight the somewhat blurred separation between the two possibilities. Apparently, the designation of such electrolytes (whether liquid or SPE apparently does not make a difference; confusion reigns everywhere) is a matter of confusion. Presumably, a statement as preferred in this report, “an SPE with added redox system”, is most reasonable whereas many of the designations suggested elsewhere like “redox-mediated” in [496] simply do not make sense and actually illustrate ignorance regarding the actual meaning of, e.g., “mediated”.

The bromine/bromide redox system has been used in a device with a PVA-based gel electrolyte with Na_2_SO_4_ and the ionic liquid *N*-butyl-*N*-methylpyrrolidinium bromide, which also supplies the bromide ions of the redox system [497]. The electrolyte composition enabled an increased cell voltage of up to 2 V. The available energy density increased accordingly. Self-discharge, frequently addressed as a possible weakness in supercapacitors with added redox systems [31], is comparable to the values recorded for the same device without the added redox component at least in the initial four hours. After 8000 cycles, 81% of the initial capacitance was still present; without the redox component, only 58% were kept. A similar approach was pursued by adding Li_2_SO_4_ and 1-butyl-3-methylimidazolium bromide (the source of the bromide/tribromide redox system) to PVA [498]. Moreover, 88% of the initial capacitance was left after 10,000 cycles; the low self-discharge as well as the relatively high stability were attributed to the use of carbon nanotubes added into the PVA.

A SPE of PVDF-HFP with an entrapped IL and 1-butyl-1-methylpyrrolidinium bromide as part of the subsequently established redox system^24^ was used in a supercapacitor with 26% capacitance loss after 10,000 cycles [499]. To an SPE of PVDF-HFP with adiponitrile as a plasticizer and an IL, diphenylamine and copper iodide were added as redox components [500]. Between two activated carbon electrodes, a supercapacitor was formed, which kept less than 6% of its initial capacitance after 5000 cycles. PVA with H_2_SO_4_ as GPE containing KI for increased storage capability has been tested in a redox capacitor with PEDOT electrodes [501]. Moreover, 74% capacitance retention was found after 1000 cycles. PVA combined with an IL and added NaI as a redox system was used as SPE in a supercapacitor with no stability data reported [502]. An SPE of PVA with H_3_BO_3_ and added KI as a redox component has been tested in a supercapacitor; stability was not revealed [503].

With an SPE based on PEO with LiClO_4_ as an ion source and an added NaI and I_2_ as a redox system, a divided supercapacitor with a Nafion^®^ 117 separator was tested; 90% of the initial capacitance was still presented after 3000 cycles [504]. Why this device is mediator-enhanced remains a mystery.

A divided cell with a separator of PVDF and LiTFSI (prepared by mixing of respective powder, dissolution in acetone, and evaporation of the solvent) and NaI/I_2_ added into a PEO/LiClO_4_ SPE has been reported [505]. Stability was not examined; the term mediator was certainly used incorrectly.

PVA with Li_2_SO_4_ as an ion source and 1-butyl-3-methylimidazolium iodide as the redox couple yielded an SPE test in a supercapacitor keeping 81% of its initial capacitance after 10,000 cycles [506].

An optimized mixture of iota carrageenan and acacia gum plasticized with ethylene glycol with added LiI^25^ as the redox-active component has been tested in a device [507]. Whether the device should be called symmetric—suggesting that the additional redox proceeds at both electrodes—appears to be dubious. The power density stays constant along 250 cycles, whereas energy density drops to about 1/100 during these cycles!

The authors repeated the approach but used LiClO_4_^26^ as an ion source and used the obtained SPE in an EDLC device [508]. Capacitance retention was not reported; gravimetric energy density decreased stepwise to less than the initial value during 1000 cycles; power density stayed constant during this test.

Although the PVA-based electrolytes with different pH values in the device with a Nafion^®^-membrane as a separator are not redox-active as claimed in the title, the added LiBr provided additional charge storage at the positive electrode [509]. The different pH values, acidic at the positive and neutral at the negative electrode, provided advantages at the price of the additional separator. Moreover, 93% of the initial capacitance was left after 10,000 cycles.

A further variation of a divided supercapacitor with different redox additives in the two electrode compartments has been reported [18]. To the PVA/H_2_SO_4_ GPE employed in both halves separated by a Nafion^®^ 117 membrane, hydroquinone (positive electrode) or methylene blue (negative electrode) was added. Moreover, 90% of the initial capacitance was still present after 3000 cycles. With the same divided cell arrangement and SPE but different redox components, VOSO_4_ (positive electrode) and Na_2_MoO_4_ (negative electrode), a supercapacitor keeping 80% of its initial capacitance after 3000 cycles was assembled [510].

A “water-in-salt” hydrogel electrolyte prepared by dissolving KBr (source of the redox-active constituent) and PVA in a 5 m solution of LiTFSI was used in an EDLC device [12]. As opposed to the author’s claim that the electrolyte is not redox-active, only the bromide/tribromide redox couple shall be called as such. The addition of this redox couple resulted in doubling the capacitance; without it, capacitance retention with growing current density is poorer with redox couple, which is possible because of incomplete redox conversion of material deep inside the porous electrode. After 5000 cycles, about 80% of the initial capacitance was still present.

Tris(ethylenediamine) cobalt(III) chloride was added as a redox component to a PVA/H_2_SO_4_ GPE, yielding a supercapacitor with 96% capacitance retention after 1000 cycles [511]. Self-discharge—the omnipresent problem with this type of storage system—was mentioned in the introduction of the report but not even touched upon in the investigation or the further report. CoSO_4_ has been added to a poly (acrylic acid)-based SPE, which showed in a complete device a 7% loss of its initial energy after 10,000 cycles; capacitance retention was not reported [512].

VOSO_4_ was added to a PVA-H_3_PO_4_ SPE; the SPE was tested in a flexible fibrous supercapacitor, showing 92% capacitance retention after 5000 cycles [513].

To a PVA-KOH SPE, K_3_Fe(CN)_6_ has been added as a redox component, providing a supercapacitor with 95% capacitance retention after 500 cycles [514]. A divided cell with a separator of PVDF and LiTFSI (prepared by mixing of respective powder, dissolution in acetone, and evaporation of the solvent) and K_3_Fe(CN)_6_/K_4_Fe(CN)_6_ added into a PEO/LiClO_4_ SPE has been reported [505]. Stability was not examined. A SPE prepared by dissolving PVDF in acetone LiTFS was used as SPE in an asymmetric supercapacitor with one carbonaceous electrode, and one such electrode with added Prussian Blue (K_2_Fe^II^Fe^III^(CN)_6_) was tested; after 5000 cycles, 93% of the initial capacitance was left [515]. Into a self-healing cross-linked double network of poly-acrylic acid/polyisodecyl methacrylate as SPE K_3_Fe(CN)_6_ was added, yielding a device showing 75%^27^ capacitance retention after 4000 cycles [516]. These authors tried another polymer mixture using PAA (presumably polyacrylic acid) and stearyl acrylate with H_3_PO_4_ as SPE with NH_4_VO_3_ and FeSO_4_ as redox components and obtained a supercapacitor, which kept 79% of its initial capacitance after 1200 cycles [517]. ZnSO_4_ in a PVA/H_2_SO_4_ served as a redox component in a supercapacitor, which showed 73% capacitance retention after 5000 cycles [518]. A blend of PVA-PVP as SPE in a supercapacitor with copper nanoparticles added into the graphite electrodes as a redox component has been reported [519].

The addition of redox-active phosphomolybdic acid or sodium molybdate to a mixture of PVA and H_3_PO_4_ yielded a material that was applied to carbon paper electrodes [520]. After evaporation of excessive water, the electrodes were assembled with porous dialysis membranes as separators. A mix of both molybdenum compounds yielded the best results; after 1000 cycles, 63% of the initial capacitance was left. The use of this GPE with phosphomolybdic acid has been suggested; the device kept 50% of the initial capacitance after 2000 cycles [503]. A PVA-based SPE with Na_2_SO_4_ added GO for improved ion conductance, and as redox systems, Na_2_MoO_4_ or NiMoO_4_ has been used in a supercapacitor [521]. The highest specific capacitance was recorded with added NiMoO_4_; the device kept 85% of the initial capacitance after 1000 cycles. To an SPE of poly (2-acrylamido-2-methyl-1-propanesulfonic acid) ammonium molybdate was added as a redox system yielding a capacitor keeping 92% of its initial capacitance after 2500 cycles [522].

A poly (vinylphosphonic acid) hydrogel with ammonium molybdate as a redox component enabled a supercapacitor to keep the optimum composition of the SPE at about 66% of its initial capacitance after 2500 cycles [523].

A SPE based on methyl cellulose with added BMITFSI with various polyoxometalates as redox systems have been studied [524]. Depending on the type of redox system, capacitance losses, ranging from 20 to 40% after 10,000 cycles, were found. Nanofibrillar methyl cellulose with an encapsulated IL was suggested as an SPE [525].

A composite of the redox system methyl orange (MO) with reduced graphene oxide as electrode has been combined with an aqueous gel electrolyte of PVA and sulfuric acid has been studied [526]. After a capacity loss of 32% in the first 2500 cycles, the capacitance stayed stable over an unspecified number of further cycles. This approach apparently is different in principle from the initial concept of the redox system dissolved in the electrolyte solution. Actually, it is much closer to a supercapacitor electrode based on charge storage by redox reactions than simple double-layer charging. MO is soluble in water; its behavior in the water-based electrolyte is not addressed in the report. A hint at lower power density may suggest a higher ESR caused by the gel electrolyte instead of an electrolyte solution; unfortunately, the quoted reference is utterly misleading.

**Figure 17:**
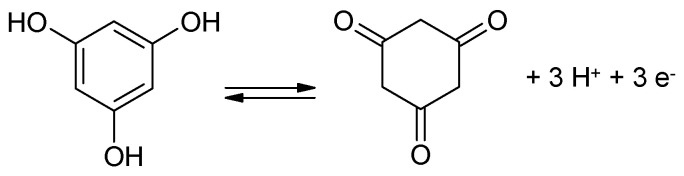
Redox reaction of phloroglucinol.

Phloroglucinol (Figure 17) was added as a redox component to a PVA and LiClO_4_,yielding a device with 94% capacitance retention after 5000 cycles but only 10% added specific capacitance due to the phloroglucinol [527].

**Figure 18:**
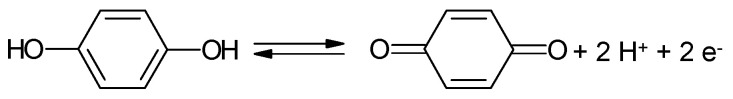
Redox reaction of hydroquinone.

To an electrolyte of PVA with KOH, hydroquinone (Figure 18) was added as a redox-active component, yielding a supercapacitor with 84% capacitance retention after 1000 cycles [528].

A blend of PVA and PVP with an IL was used as an SPE with hydroquinone as redox components added; the supercapacitor had a stable capacitance along 5000 cycles [529]. A similar combination of PVA, with H_2_SO_4_ as an SPE, and *p*-benzenediol^28^ as the redox-active component has been examined [530]. A specific capacitance, slightly depending on the operating voltage range of the device, was reported; along 3000 cycles, about 9% of the initial capacitance was lost. To an SPE of PVDF-HFP with an IL and a “plastic crystal succinonitrile” hydroquinone was added as redox component [531]. The assembled supercapacitor kept 55% of the initial capacitance after 10,000 cycles.

Further increases in specific capacitance may become possible when combining redox electrodes with electrolytes containing redox systems. Care must be exercised in such combinations when matching redox potentials where redox transformations inside the electrodes and in the electrolyte proceed. The highest capacitance values were obtained with composite electrodes of polyaniline and MCNTs combined with a solid electrolyte of PVA, and sulfuric acid contained hydroquinone [532]. The device kept 89% of its initial capacitance after 2000 cycles. Corn starch has been combined with H_3_PO_4_ into an SPE with added hydroquinone as a redox component [533]. The assembled supercapacitor kept 87% of the initial capacitance after 10,000 cycles. Oligomeric 1,5-diaminoanthraquinone^29^ prepared by electrodeposition on a carbon support and combined with either a PVA–H_3_PO_4_ blend or PMMA–PC–EC–TEAClO_4_ SPE in a symmetric redox capacitor [534]. The latter device showed somewhat better stability: After an initial drop of capacitance by about 50% after 1400 cycles, even less was retained. Why only the latter electrolyte was called a GPE is as mysterious as the reason for assembling two identical redox electrodes with their operating electrode potential fixed by the ongoing redox process. The latter question has been addressed in detail before [38]; nevertheless, such arrangements have questionable popularity.

**Figure 19:**
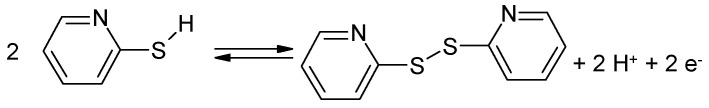
Redox reaction of 2-mercaptopyridine.

A supercapacitor with added 2-mercaptopyridine (Figure 19) as a redox-active material (for unknown reasons, the author calls this redox-mediated) and PVA-phosphoric acid electrolyte and two AC electrodes have been described [535]. The redox compound elsewhere studied with respect to its capability to form self-assembled monolayers [536,537] undergoes a dimerization redox reaction according to [538,539,540].

The added redox system offers an increase of the energy density to about five times the value recorded in its absence; it is better than some previously studied systems, which are mostly based on a PVA-KOH electrolyte. Somewhat disturbingly, several values quoted as a comparison in the report are even higher, and not smaller! After 1000 cycles, about 80% of the initial capacitance is retained. The same approach was tried with a PVA-H_2_SO_4_ SPE with 89% capacitance retention after 3000 cycles [541].

**Figure 20:**
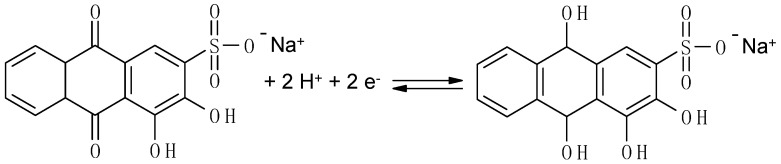
Redox reaction of alizarin red S.

Indigo carmine added into a PVA-H_2_SO_4_ electrolyte resulted in ionic conductivity by a factor of two (presumably because the dye molecule has two sulfonate groups) and a doubling of the specific capacitance [542]. Moreover, 80% of the initial capacitance is left after 3000 cycles. A similar concept has been realized with the redox-active dye alizarin red S [543]. The anionic dye molecule (see Figure 20) provides additional ionic conductivity.

**Figure 21:**
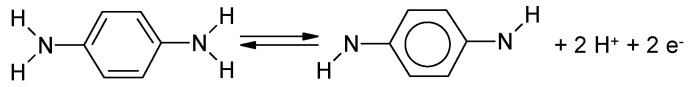
Redox reaction of *p*-phenylenediamine.

Unfortunately, and for reasons not reported, capacitance retention was significantly poorer than with indigo carmine; only 78% of the initial capacitance was left after 1000 cycles. In a divided cell (Nafion^®^ separator), alizarin red S and *p*-phenylenediamine (see Figure 21) were added to the alkaline (PVA + KOH) electrolyte solution on the positive half-cell, the negative half-cell used the same electrolyte and a metal (Co, Ni) oxide electrode [544]. The device kept 94% of its initial capacitance after 4000 cycles. *p*-phenylenediamine was added to an SPE of PVA and KOH as a redox-active component [545]. A device assembled with two activated carbon electrodes kept 83% of its initial capacitance after 1000 cycles. Self-discharge or chemical follow-up reactions of the radical intermediates formed by electrooxidation of the diamine were not addressed.

Methylene blue (MB) was added as a redox-active component to a GPE made of a blend of PVA and PVP with H_2_SO_4_ [546]. The addition of MB almost tripled the storage capability, which stayed at 91% of the initial value after 2000 cycles. A similar study yielded elsewhere 36% capacitance fading after 10,000 cycles [16].

A device with two carbonaceous electrodes and a GPE prepared from soy protein isolate (a renewable resource) and Li_2_SO_4_ with added KI as a redox system provided 58% capacitance retention after 1500 cycles [547]. An electrolyte, which was prepared by dissolving PVDF-HFP in acetone and adding an ionic liquid and redox-active materials KI and diphenylamine, was drop cast on activated carbon electrodes. After drying, the electrodes were assembled with their electrolyte-coated sides facing each other [548]. In cells with only one redox component, redox processes of the iodide/triiodide redox couple and the diphenylbenzidine formed by irreversible dimerization of diphenylamine were verified, although the displayed CVs of the two-electrode cell arrangements hardly support this. The subsequently formulated claim that diphenylamine (and not as before diphenylbenzidine) establishes redox couples for charge storage in a cell with both redox components casts further doubt. Which redox reaction happens at which electrode is left to the reader’s guesswork. The somewhat diffuse description of the results of stability studies suggests about 30% capacitance loss during 6000 cycles. Self-discharge was not mentioned.

To cross-linked polyacrylamide, 1,4-butanediol diglycidyl ether CoSO_4_ (whether it is a modification or doping seems to be unclear for the authors) was added [549]. Without the added redox system, an EDLC device with AC electrodes was established and, additionally, yielded an increase of the capacitance by a factor of five. Recorded CVs were called semi-rectangular (whatever that means) without an indication of the expected redox activity. Thus, the authors concluded that the increase was due to the increased ionic conductivity of the electrolyte solution. Unfortunately, conductivity was not measured; in addition, conclusions are hampered by the vastly different electrode potential ranges in the CVs. After 10,000 cycles, 91% of the initial capacitance was still present.

The copolymer PVDF-HFP dissolved in acetone was mixed with BMITFSI (how NaI was added remains unknown) and was used as an SPE for a supercapacitor showing 5% capacitance loss after 10,000 cycles [550].

Poly (vinylphosphonic acid) combined with nickel nitrate yielded a hydrogel, which was combined with activated carbon electrodes into a device [551]. At optimum nickel content, capacitance retention of 84% after 5000 cycles was recorded. Terminology in the report appears to be confusing: The polyelectrolyte is not doped with metal but metal ions, it is hardly energized by a hydrogel, and redox-activated seems to be an unknown term.

### 3.6. Approaches Toward Improved Electrolyte/Electrolyte Interfaces

A fundamental problem always encountered with solid electrolytes and porous electrodes is the establishment of a stable and sufficiently large, extended electrode/electrolyte interface. Because the actual available capacitance and, thus, storage capability of a supercapacitor depends on the extent of the interface area, a maximum surface area and utilization of the electrode surface are of utmost importance. With liquid electrolytes and sufficient wetting, good utilization is almost natural [354]. Sometimes, a few charge/discharge cycles are needed to establish full contact and wetting after the assembly of a supercapacitor [355].

With solid electrolytes, the situation changes completely. Smooth electrodes like lithium metal foils can perhaps be brought into contact with a solid electrolyte by gentle pressing; for a porous electrode, this approach appears to be rather useless. High pressure may destroy the porosity; in addition, the penetration of the electrolyte into the porous structures seems to be highly unlikely. Nevertheless, attempts to apply only mechanical force by, e.g., using a roller press to laminate the nickel foam-based activated carbon electrodes with the GPE have been reported [552]. The device thus obtained and kept 94% after 10,000 cycles. Various options strongly depending on the type of electrolyte have been tried; sometimes, they can only be deduced from the experimental descriptions (the listed examples are discussed in detail with performance data elsewhere in the text):

- The dissolved electrolyte (polymer(s), plasticizer, electrolyte salt) is coated onto the porous electrode. Before/after drying, electrodes are assembled with/without an additional separator. Typical examples: copolymer electrolyte [119], polymer-ionic liquid mixtures [254]. Or, conversely, the electrodes are soaked in the still liquid SPE; for typical examples, see [314,344].
- In a somewhat similar procedure, some electrolytes can be electrodeposited directly on the electrode, providing immediately good interfacial contact [388] or can be formed by UV-light polymerization of the reactant solution soaked into the electrode [404].
- The electrodes are soaked with a solvent; it is possible that the same is also used in the preparation of the electrolyte; the wet electrodes are joined with the electrolyte. Transfer of ions from the electrolyte into the solvent filling the porous electrode body proceeds. Typical example: dimethylacetamide [115].
- The electrodes are soaked with monomers (mostly in suitable solvents) of the electrolyte polymer; these mono- or oligomers can even act as binders for the active electrode material. Upon assembly of the electrodes with the polymer electrolyte, a continuous transition from the polymer in the electrolyte to the monomer in the electrolyte is established. Typical example: RuO_2_ and Nafion^®^ [471].
- A liquid mixture containing SPE precursors, i.e., monomers, is soaked into the porous electrodes, and subsequently, polymerization is performed in situ. Examples: Zinc-ion capacitor [553], see also [429].
- In a similar approach to GPEs, the porous electrodes are immersed into the electrolyte mixture, which is still rather liquid. Typical example: PVA with Li_2_SO_4_ in an EDLC device [356].
- The liquid part of, e.g., a gel electrolyte, in most cases a solvent and a salt, is soaked into the porous electrode before device assembly. Typical examples: [402,407,461].
- Direct deposition of the electrode material on the SPE film. Typical examples: [308,554]. In the latter example, ultra-long CNTs were directly deposited from the gas phase aiming at a transparent supercapacitor, which finally showed a 94% capacitance retention after 20,000 cycles.

Another approach toward improved interfacial contact by forming an ionogel in situ during device preparation has been proposed [555]. Poly (vinylidenefluorideco-hexafluoropropylene) dissolved in DMF with an added IL and Al(Tf)_3_ yielded a gel-like solid electrolyte [277]. An EDLC device prepared with the electrodes soaked first with the polymer solution before assembly showed a very stable capacitance for 50,000 cycles.

An EDLC-supercapacitor prepared with a freestanding film of a mixed polymer subsequently soaked with an acetonitrile-based electrolyte solution kept 85% of its initial capacitance after 5000 cycles [90]. With some of the polymer used for film preparation dissolved in the electrolyte solution, an even better-performing device was obtained; solvent evaporation was significantly diminished at an only slightly reduced capacitance.

A different approach with the insertion of a thin layer (450 nm) based on porous TiO_2_ sprayed on the SPE has been proposed [556]. In a redox supercapacitor, a substantial capacitance increase was found. Improved performance was attributed to decreased interfacial resistance and enhanced ion transfer.

The problem of establishing an adequate electrode/electrolyte interface with GPEs in supercapacitors was addressed as early as 1998 [48].

### 3.7. Polymer Electrolytes and Device Properties

The mechanical properties of a supercapacitor beyond questions of safety and leakage are hardly discussed and considered in academic research, providing a starting point for the development of non-liquid electrolytes. Because supercapacitors (like batteries) may occupy a significant amount of space and volume in a vehicle, their integration into the mechanical structure of vehicles has been suggested; in addition, their use as construction (i.e., load-bearing) elements has been proposed. Poly (ethylene glycol) monomethyl ether acrylate with an added IL and functionalized nanosized silica filler has been tested as an SPE in a structural EDLC supercapacitor showing 91% capacitance retention at best after 100 (!) cycles [557]. For overviews, see [558,559]. Once a supercapacitor becomes a structural element and building block, solid electrolytes in a supercapacitor are essential [147,390,560,561,562,563,564,565,566,567,568,569,570,571,572,573,574,575,576,577,578,579,580,581,582,583,584].

A polymer electrolyte suitable for inkjet printing followed by UV-light curing for microsupercapacitors has been described [585].

Polymer electrolytes are essential for flexible supercapacitors; overviews are available [586,587]. Flexible solid-state supercapacitors utilizing 2D materials may require matching electrolytes; an overview has been provided [588]. Many polymers and polymer electrolytes are flexible; this property has been stressed in some reports, which are discussed above. This also applies to the use of SPE in actuators [589].

A polyampholyte saturated with simulated body fluid has been suggested as SPE for a supercapacitor in implantable electronic medical devices, showing a stable capacitance along 8000 cycles [590].

A KOH gel electrolyte (?) has been used in an EDLC device [591]. Presumably, the PVA-KOH SPE described in [592] was meant. A sodium ion-conducting GPE of secret composition^30^ was used in [593]. The initial capacitance decreased to 70% of its initial value after 1000 cycles.

Following the overlap between supercapacitor and secondary battery concepts already addressed in the introduction, examples of using gel electrolytes in devices combining battery and supercapacitor electrodes (hybrid devices) have been reported [594].

Fire hazards of supercapacitors initially associated with organic solvents (see above) are somewhat diminished with the various types of electrolytes surveyed in the preceding report. Further protection may be achieved by using fire-retardant electrolytes like in [595]. The relevant properties can be introduced by using, e.g., bromine as a substituent.

An inorganic gel polymer electrolyte based on SiO_2_ with a confined mixture of two Ils has been reported [596]. Attapulgite has been used in the preparation of a hydrogel; its use in a device with a PVA-Na_2_SO_4_ SPE also employed remains mysterious [597].

## 4. Conclusions, Outlook, and Perspectives

Major progress has been achieved with solid electrolytes, in particular with gel electrolytes of widely varying chemical composition obtained either by the gelling of liquid electrolyte solutions using a wide variety of gelling agents or by the gelling (plastification) of solid polymers with many different solvents. The number of obtained materials, with conductivities surpassing those of liquid systems, is growing. A detail frequently overlooked may be the option to assemble cells without an extra separator, which may help to reduce the actual cell resistance further.

Since the first reports on non-liquid electrolytes appeared, further aspects, which are mostly related to environmental considerations, have become the focus of attention. Accordingly, systems without fluor-containing components but with biopolymers from renewable sources, instead, enjoy more and still growing attention.

For practical and even commercial applications, the stability of the supercapacitor, both of EDLC- and redox-type assembled with the developed electrolytes, is of major interest. Surprisingly, low interest has been devoted to this major aspect in many reports. Future studies should pay more attention to this aspect.

## Notes

1 Possibly the authors also know somebody who is quasi-dead.

2 This acronym is used throughout this report without repeating the imprecise definition again and again.

3 TFSI is the common acronym designating the IL-anion bis(trifluoromethanesulfonyl)imide.

4 A reference provided in this report turns out to be completely unrelated and useless.

5 To call the device a magnesium capacitor apparently only because a magnesium salt was added seems to be a far stretch of the facts.

6 Assuming the acronym CMC nowhere explained in this report has this meaning.

7 Different from the author’s opinion this is the name of the plant, not of any chemical compound!

8 Presumably the term “cycle retention” means capacitance retention.

9 In this report the discussion quotes a non-existent report on ion transport attributed to Vehicle and Grotthus—a most memorable error in this also otherwise flawed report.

10 The synonym plastified is also found.

11 The terms gel and gelled are sometimes mixed up, confused and taken as synonyms–although the former is a noun, the latter an adjective. Gel’s can be obtained by gelling of a liquid by adding a gelling agent [2], they can also be obtained by treating a solid, e.g. a polymer, with a suitable liquid. This process is also called plastification because it yields a more „plastic“ material. Both materials may behave similar and may have similar properties, accordingly the distinction is sometimes difficult. In this report assignments and claims of the authors are used as points of reference whenever possible.

12 Tf or TF are common acronyms specifying the IL-anion trifluoromethanesulfonate.

13 This acronym is not exactly systematic; it misses the -co- between the two constituents, it also misses the P with HFP. Nevertheless, it appears to be firmly established, and unlike with some other authors (see, e.g., the systematically speaking more reasonable P(VDF-HFP) in [201]), no attempt is made to create a new and finally only confusing further acronym. The acronym PVDF(HFP), as used in [202,203], definitely makes no sense. Sometimes, the authors apparently also accepted this fact; elsewhere in the report, they call the copolymer PVDF-HFP. The acronym PVdF(HFP) also lacks logic [204].

14 Why this SPE has been called SN-based is mysterious at least.

15 For mysterious reasons these authors coined the rather non-logical acronym HEP.

16 Only one of numerous inconsistencies in this report.

17 The report is hard to understand and lacks many relevant details.

18 The authors of this study conveniently ignore completely an earlier study with exactly this electrolyte system in [33].

19 Why this material is a biopolymer remains as unclear as the biodegradability remains questionable given the IL-content.

20 Why this SPE can be reasonably called “ionic liquid based” remains mysterious.

21 MWNT appears to be a rather uncommon acronym used only by these authors.

22 To assign the acronym CNF, which is used to name carbon nanofibers in the rest of the world, to this material is a most inspired idea to mislead readers.

23 Certainly this material does not contain CF_3_ chains as claimed by the authors in [462].

24 The electrolyte itself is hardly redox-active as claimed in the report’s title.

25 The material is certainly not LiI-based as claimed in the title of the report.

26 Certainly this is not lithium perchloride as stated in the report.

27 Whether this shall be called outstanding seems to be a matter of further debate.

28 The scientific community calls this compound hydroquinone.

29 Solubility of this oligomer is not addressed in the report. In case it is really completely insoluble this system is not properly placed in this section.

30 Close examination of supplementary information revealed it as PVDF-HFP dis-solved in DMF; the obtained membrane was soaked in a solution of NaPF_6_ in ethylene carbonate and dimethyl carbonate.
